# EspFu-Mediated Actin Assembly Enhances Enteropathogenic *Escherichia coli* Adherence and Activates Host Cell Inflammatory Signaling Pathways

**DOI:** 10.1128/mBio.00617-20

**Published:** 2020-04-14

**Authors:** Fernando H. Martins, Ashwani Kumar, Cecilia M. Abe, Eneas Carvalho, Milton Nishiyama-Jr, Chao Xing, Vanessa Sperandio, Waldir P. Elias

**Affiliations:** a Laboratory of Bacteriology, Butantan Institute, São Paulo, Brazil; b Department of Microbiology, University of Texas Southwestern Medical Center, Dallas, Texas, USA; c Department of Biochemistry, University of Texas Southwestern Medical Center, Dallas, Texas, USA; d Eugene McDermott Center for Human Growth and Development, University of Texas Southwestern Medical Center, Dallas, Texas, USA; e Laboratory for Applied Toxinology, Center of Toxins, Immune-Response and Cell Signaling (CeTICS), Butantan Institute, São Paulo, Brazil; f Department of Bioinformatics, University of Texas Southwestern Medical Center, Dallas, Texas, USA; g Department of Population and Data Sciences, University of Texas Southwestern Medical Center, Dallas, Texas, USA; University of Georgia

**Keywords:** enteropathogenic E. coli, EspFu/TccP, Tir phosphorylation, pedestal formation, inflammation

## Abstract

The translocation of effectors into the host cell through type 3 secretion systems (T3SS) is a sophisticated strategy employed by pathogenic bacteria to subvert host responses and facilitate colonization. Enteropathogenic Escherichia coli (EPEC) and enterohemorrhagic E. coli (EHEC) utilize the Tir and EspFu (also known as TccP) effectors to remodel the host cytoskeleton, culminating in the formation of attaching and effacing (AE) lesions on enterocytes. While some EPEC strains require tyrosine phosphorylation of Tir and recruitment of the host Nck to trigger actin polymerization, EHEC and certain EPEC strains, whose Tir is not phosphorylated, rely on the effector EspFu for efficient actin remodeling. Here, we investigated the role played by Tir-Nck and Tir-EspFu actin polymerization pathways during the infection of epithelial cells, as well as the host transcriptional response to the AE lesion formation induced by EPEC. We found that EspFu-mediated actin assembly promotes bacterial attachment and epithelial colonization more efficiently than Tir-Nck. Moreover, we showed that both actin polymerization mechanisms can activate inflammatory pathways and reverse the anti-inflammatory response induced by EPEC in epithelial cells. However, this activity is remarkably more evident in infections with EspFu-expressing EPEC strains. This study demonstrates the complex interactions between effector-mediated actin remodeling and inflammation. Different strains carry different combinations of these two effectors, highlighting the plasticity of pathogenic E. coli enteric infections.

## Abstract (precis)

EPEC is among the leading causes of diarrheal disease worldwide. The colonization of the gut mucosa by EPEC results in actin pedestal formation at the site of bacterial attachment. These pedestals are referred to as attaching and effacing (AE) lesions. Here, we exploit the different molecular mechanisms used by EPEC to induce AE lesions on epithelial cells, showing that the effector EspFu is associated with increased bacterial attachment and enhanced epithelial colonization compared to the Tir-Nck pathway. Moreover, we also showed that actin pedestal formation can counterbalance the anti-inflammatory activity induced by EPEC, especially when driven by EspFu. Collectively, our findings provide new insights into virulence mechanisms employed by EPEC to colonize epithelial cells, as well as the host response to this enteric pathogen.

## INTRODUCTION

Enteric bacterial pathogens have evolved specialized infection strategies to manipulate host signaling pathways and facilitate the colonization of the gastrointestinal tract. Many of these pathogenic bacteria utilize the T3SS to translocate a repertoire of effector proteins into the host cell cytosol (1). These effectors, in a coordinated fashion, modulate several cellular processes, such as cytoskeletal dynamics, membrane trafficking, transcription, cell cycle progression, signal transduction, and ubiquitination (2).

Enteropathogenic Escherichia coli (EPEC) is one of the leading causes of diarrheal diseases worldwide, especially among children younger than 5 years of age (3, 4). Similarly to other attaching-effacing (AE) pathogens (e.g., enterohemorrhagic E. coli [EHEC] and Citrobacter rodentium), EPEC employs effector proteins to remodel the host cytoskeleton, enabling its adherence to enterocytes and colonization of the intestinal epithelium. The cytoskeletal rearrangement promoted by EPEC culminates in AE lesions, which are characterized by the effacement of the microvilli, and the accumulation of polymerized actin beneath the attached bacteria, forming pedestal-like structures (5).

The majority of the genes required for AE lesion formation are contained within a pathogenicity island named the locus of enterocyte effacement (LEE) (6). The LEE region is organized into five major operons (*LEE1* to *LEE5*), which encode the structural components of the T3SS (7), effector proteins (8), regulators, chaperones, the adhesin intimin (8), and its translocated receptor Tir (9). Tir is inserted into the eukaryotic membrane and phosphorylated by host cell tyrosine kinases at the C-terminal domain (at tyrosine Y_474_, yielding Tir_Y-P_) after its interaction with intimin (10). Following phosphorylation, the mammalian protein Nck is recruited, where it activates the neural Wiskott-Aldrich syndrome protein (N-WASP), which in turn recruits the Arp2/3 complex, leading to a localized actin polymerization (11). In contrast, Tir from some EPEC and EHEC strains lacks the Y_474_ and is not tyrosine phosphorylated (Tir_S_). Instead of recruiting Nck, Tir_S_ engages the non-LEE-encoded effector EspFu/TccP to promote pedestal formation (12, 13). EspFu is also translocated to the host cell by the LEE-encoded T3SS, where it bridges the interaction of Tir with the host IRSp53 and/or IRTKS proteins and N-WASP. A conserved Asn-Pro-Tyr motif (NPY) in Tir is a critical binding site for the I-BAR domain of IRSp53 and IRTKS (14), while the SH3 domain of these proteins directly binds to the C-terminal proline-rich region of EspFu (15). EspFu then interacts with the GTPase-binding domain (GBD) to activate N-WASP (12), recruiting the Arp2/3 complex and leading to actin polymerization. In addition, a subset of EPEC strains has the potential to induce actin polymerization by simultaneously utilizing the Tir-Nck and Tir-EspFu pathways (16–19). However, it is unclear whether the ability to use converging pedestal formation pathways would confer a competitive advantage for these strains or whether host epithelial cells respond differently to these distinct actin polymerization pathways.

EPEC also encodes a vast repertoire of non-LEE-encoded effectors that are not directly involved with cytoskeleton remodeling but instead play an important role in the modulation of host inflammatory response during the course of infection (20–27). These effectors are highly conserved among AE pathogens and are generally encoded by genomic islands, suggesting that the acquisition of these elements was necessary to attenuate the inflammatory response triggered by EPEC’s interaction with the host cell. In fact, the attenuation of the inflammatory response triggered by AE pathogens is essential to establish a suitable niche for host colonization. For example, mutant strains of C. rodentium that are not able to suppress inflammation had lower murine intestinal colonization compared to wild-type strains (28). However, how this immunomodulation is impacted by the different actin polymerization mechanisms employed by EPEC to induce AE lesions is still poorly understood.

In this study, we investigated the different molecular mechanisms used by EPEC to induce AE lesions on epithelial cells, showing that the effector EspFu is associated with increased bacterial attachment and enhanced epithelial colonization compared to the Tir-Nck pathway. Moreover, we also show that actin pedestal formation can counterbalance the anti-inflammatory activity induced by EPEC, especially when driven by EspFu.

## RESULTS

### The effector repertoire of EPEC BA320 strain.

Usually, EHEC strains employ the Tir_S_-EspFu pathway (12, 13) and EPEC strains use the Tir-Nck pathway (11) to form AE lesions. BA320 is an EPEC O55:H7 strain that was isolated from the stool of a child with acute diarrhea in 2003 during an epidemiological study focused on the etiology of acute diarrhea in children under 5 years old carried out in Salvador, Brazil (29). The BA320 strain was previously reported to have the potential to induce actin polymerization in cultured epithelial cells *in vitro* (30). Importantly, EPEC O55:H7 gave rise to EHEC O157:H7 (31).

We sequenced the genome of BA320 to better elucidate the molecular mechanisms employed by this strain to induce AE lesions, focusing on its effector repertoire. The draft genome of strain BA320 is 5,360,779 bp in size with 50.39% G+C content, and it contains 5,281 coding sequences, 7 rRNA (rrn) operons, and 87 tRNA genes. A single 65-kb plasmid was detected, which does not correspond to the EPEC adherence factor (EAF) virulence plasmid. Also, the operons encoding the bundle-forming pilus (32) and Per regulators (33) were not found, corroborating previous studies that classified BA320 as an atypical EPEC strain (30, 34).

**FIG 1:**
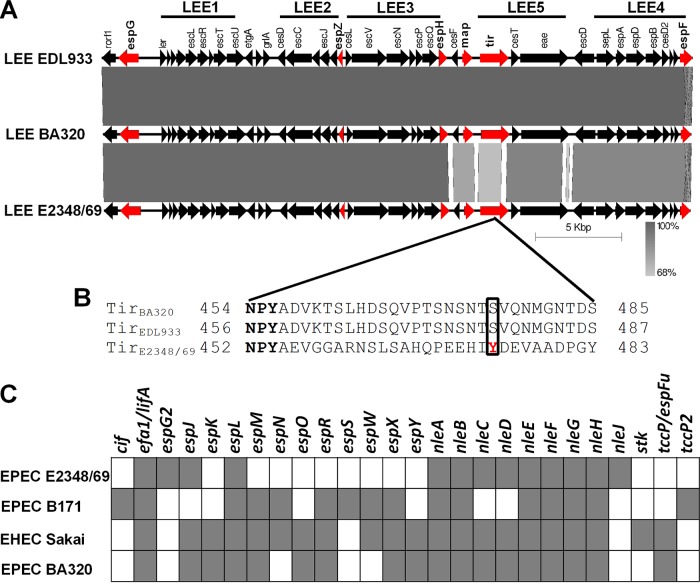
Effector repertoire of EPEC BA320. (A) Homology comparisons among the LEE regions of EPEC BA320, EHEC EDL933, and EPEC E2348/69. Effector-encoding genes are labeled in red. The size and direction of the arrows indicates the size of each predicted gene and the direction of transcription. The scale of 5 kb is indicated at the bottom. (B) Sequence alignment of the C-terminal region of Tir. The NPY motif is conserved in all three strains (in bold). Phosphorylated tyrosine (Y474) in Tir_E2348/69_ and equivalent residues in Tir_BA320_ and Tir_EDL933_ are boxed with a solid line. (C) *In silico* detection of the non-LEE-encoded effectors of EPEC E2348/69, EPEC B171, and EHEC Sakai in the EPEC BA320 genome using TBLASTN. Each square indicates the presence (in gray) or absence (in white) of an effector-encoding gene.

The LEE pathogenicity island of BA320 is 34,166 bp long showing 99 and 91% nucleotide identities with the LEE regions of prototype strains EDL933 (EHEC O157:H7) and E2348/69 (EPEC O127:H6), respectively (Fig. 1A). Multiple sequence alignment showed higher nucleotide diversity between the *LEE4* and *LEE5* operons from BA320 and E2348/69, while the *LEE1-3* operons shared higher similarity. Six effector-encoding genes (*espG*, *espZ*, *espH*, *map*, *tir*, and *espF*) are present in the LEE of BA320, of which Tir is the only one absolutely required for AE lesion formation on epithelial cells (35). Analysis of the Tir_BA320_ sequence identified the NPY motif implicated in the EspFu-mediated actin polymerization pathway in EHEC O157:H7, while the tyrosine residue Y474, required for Nck-dependent actin polymerization in EPEC O127:H6, was absent (Fig. 1B).

We next examined the presence of non-LEE-encoded effector genes in the BA320 genome by comparison to the effector repertoire of the reference strains E2348/69, Sakai (EHEC O157:H7) and B171 (EPEC O111:H–). Using this approach, we identified 36 candidate effector genes belonging to 18 different families, including *efa1/lifA*, *espJ*, *espK*, *espL*, *espM*, *espO*, *espR*, *espX*, *espY*, *nleA*, *nleB*, *nleC*, *nleD*, *nleE*, *nleF*, *nleG*, *nleH*, and *espFu/tccP* (Fig. 1C). Overall, our *in silico* analysis identified 42 potential genes encoding T3SS effectors into the BA320 genome.

### EspFu promotes actin remodeling and bacterial attachment to epithelial cells.

BA320 induces actin polymerization on epithelial cells *in vitro* in a Nck-independent manner (30). Congruently, the genome analyses indicated that an EspFu-mediated signaling pathway would be the most likely molecular mechanism employed by BA320 to induce actin polymerization. *In silico* analysis showed that the *espFu* gene is located within a prophage, which shares low similarity to the CP-933U and Sp14 prophages from strains EDL933 and Sakai, respectively (see Fig. S1A in the supplemental material). The *espFu* gene of BA320 is 732 bp long and encodes a protein containing four almost identical 47-amino-acid proline-rich repeats (PRRs) with high similarity to EspFu from EHEC O157:H7 (strain EDL933) and EPEC O119:H6 (strain ICC199) (Fig. S1B).

10.1128/mBio.00617-20.1 FIG S1 Genomic and phenotypic characterization of EPEC strains. (A) Schematic representation of the genomic localization of *espFu* in EPEC BA320 and homology comparison to the prophages CP-933U and Sp14 from strains EHEC EDL933 (top) and EHEC Sakai (bottom), respectively. (B) Multiple sequence alignment of EspFu from strains EPEC BA320, EHEC EDL933 (GenBank accession no. AAG56991) and EPEC ICC199 (GenBank accession no. ABB16333). The complete proline-rich repeats (PRRs) with 47 amino acid residues and a partial repeat in the EspFu proteins are indicated by solid arrows and a dashed arrow, respectively. Amino acids residues identical in all three proteins are indicated by shading and asterisks, while amino acids substitutions are indicated in white. (C) Growth profiles of EPEC strains in low-glucose DMEM at 37°C and 5% CO_2_, confirming that the mutagenesis procedures did not alter bacterial growth rates. The data are represented as means ± SD of two independent experiments performed in triplicates. (D) Western blot assay of EspB secreted from strains grown for 6 h in low-glucose DMEM at 37°C and 5% CO_2_ showing the T3SS functionality. Bovine serum albumin (BSA) was used as the loading control. Download FIG S1, TIF file, 0.4 MB. Copyright © 2020 Martins et al. 2020 Martins et al. This content is distributed under the terms of the Creative Commons Attribution 4.0 International license.

**FIG 2:**
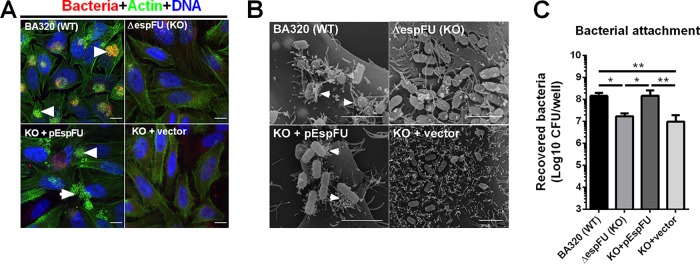
EspFu is required for efficient actin pedestal formation and increases EPEC adherence. (A) Detection of pedestal formation using the fluorescent actin staining (FAS) test on HeLa cells infected with mCherry-expressing EPEC strains (red) for 6 h. Cells were fixed and stained with FITC-phalloidin and DAPI to visualize actin (green) and DNA (blue). Pedestals were visualized as bright green structures (white arrowheads). Scale bar, 10 μm. (B) Representative scanning electron micrographs of HeLa cells infected with the indicated EPEC strains. White arrowheads point to actin pedestals. Scale bar, 5 μm. (C) Quantitative adherence tests were performed using dilution plating to count attached bacteria after lysing HeLa cells with 1% Triton X-100. Data presented are means ± standard deviations (SD) of six biological replicates. Statistical analysis was performed by one-way ANOVA, followed by a *post hoc* Tukey test. *, *P* < 0.05; **, *P* < 0.01.

While the wild-type BA320 strain (WT) is able to induce robust actin polymerization *in vitro*, the deletion of *espFu* resulted in the loss of this phenotype (Fig. 2A), which was restored by an EspFu-producing plasmid (KO+pEspFu), but not with an empty vector (KO+vector). These findings were consistent with scanning electron microscopy (SEM) analysis showing the presence of actin pedestals on the surface of HeLa cells infected with WT and KO+pEspFU but not with *espFu*-negative strains (Fig. 2B). Moreover, EspFu-producing strains were significantly more adherent than KO and KO+vector (Fig. 2C). These data indicate that EspFu plays an essential role in AE lesion formation and increases bacterial attachment to epithelial cells.

### EspFu-mediated actin assembly promotes more efficient bacterial attachment and pedestal formation than the Tir:Nck-dependent pathway in EPEC BA320.

**FIG 3:**
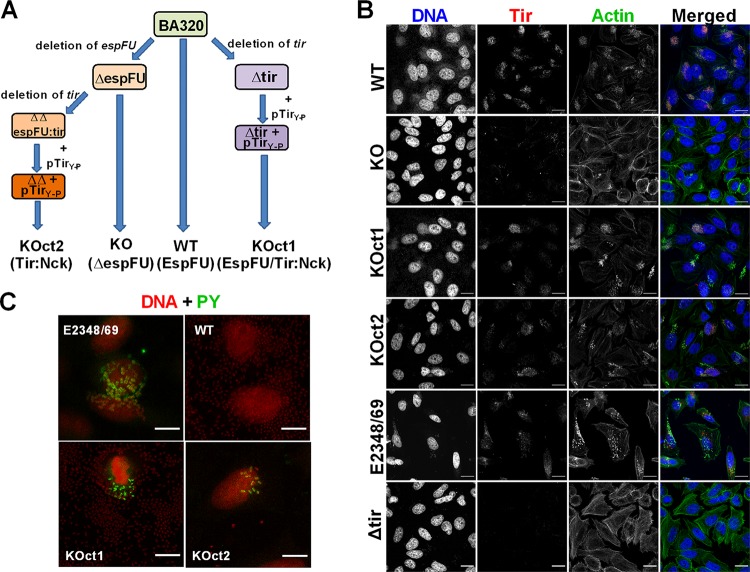
Engineering EPEC strains that display distinct molecular mechanisms to induce actin polymerization. (A) Schematic representation of the strategies employed to generate the EPEC strains used in this study. While BA320 (WT) utilizes EspFu to trigger actin polymerization, KOct2 and KOct1 were engineered to reflect the Tir-Nck and EspFu/Tir-Nck pathways of pedestal assembly, respectively. (B) Immunofluorescence assay showing production and translocation of Tir to the host cell. HeLa cells were infected with the indicated strains, fixed, and then labeled with rabbit anti-Tir polyclonal serum (red), FITC-phalloidin (actin; green), and DAPI (bacteria and cell nuclei; blue). (C) Immunofluorescence assay showing Tir tyrosine phosphorylation in cells infected with KOct1 and KOct2 strains. E2348/69 and BA320 were used as positive and negative controls, respectively. Cells were labeled with mouse anti-phosphotyrosine (PY) monoclonal antibodies conjugated with FITC (green) and propidium iodide (bacteria and cell nuclei; red). Scale bar, 20 μm.

In addition to EspFu, EPEC strains can also induce actin polymerization through the Tir:Nck-dependent pathway or even using both signaling pathways (18, 19). To compare the different actin assembly pathways employed by EPEC, we genetically manipulated strain BA320 by targeting the AE effectors Tir and EspFu, and two sets of isogenic strains were generated: (i) BA320 (WT) and Δ*espFu* (KO), and (ii) KOct1 and KOct2 (Fig. 3A). These strains differ by the presence (WT and KOct1) or absence (KO and KOct2) of *espFu*, as well as by the location and type of *tir* (i.e., chromosomal and nonphosphorylated in WT and KO; plasmid encoded and phosphorylated in KOct1 and KOct2). Specifically, BA320 represents the EspFu-mediated actin assembly; KOct2 and KOct1 strains can induce actin rearrangement via Tir-Nck or both EspFu/Tir-Nck pathways, respectively, while KO is a pedestal-deficient strain.

All these EPEC strains showed similar growth profiles (Fig. S1C) and T3SS functionality, as assessed by the secreted levels of EspB (Fig. S1D). Immunofluorescence assays showed that Tir was translocated to host cells and colocalized with pedestals formed by BA320, KOct1, and KOct2 strains (Fig. 3B). The KO translocated Tir but did not induce robust actin rearrangement. Anti-phosphotyrosine (PY) labeling revealed that Tir of both KOct1 and KOct2 strains were phosphorylated similarly to Tir from the EPEC prototype strain E2348/69 (Fig. 3C), while Tir of BA320 was not phosphorylated after translocation to the host cell, as expected. These strains were then considered suitable for comparisons of the different actin assembly pathways used by EPEC.

**FIG 4:**
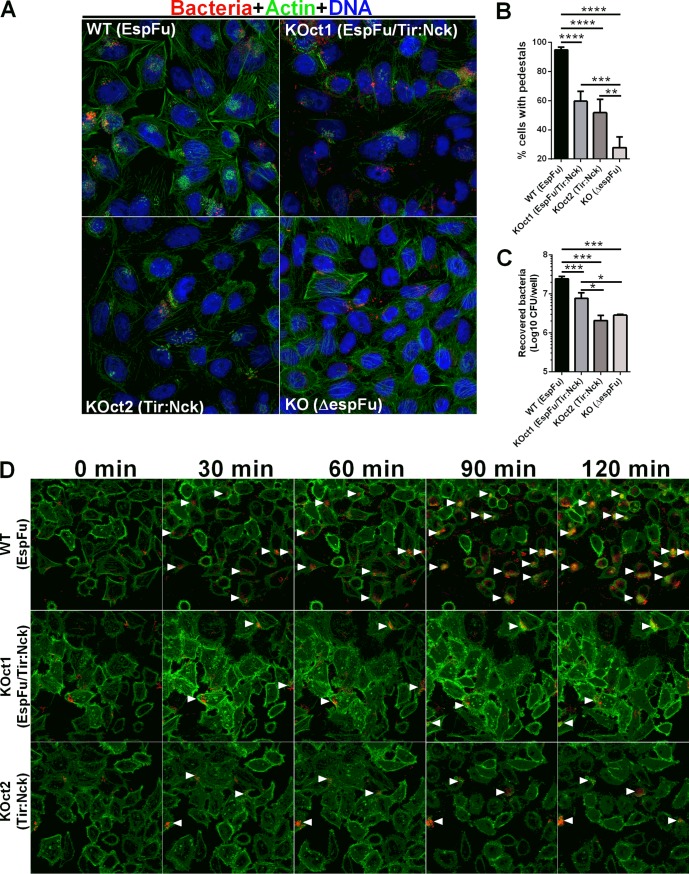
EspFu-mediated actin assembly promotes more efficient bacterial attachment and pedestal formation. (A) FAS assay for detection of pedestal formation on HeLa cells infected with mCherry-expressing EPEC strains (red) for 6 h. Actin and DNA were stained with FITC-phalloidin (green) and DAPI (blue), respectively. Original magnification, ×63. (B) Quantification of FAS showing the percentage of cells with EPEC forming actin pedestals. The number of cells with pedestals was enumerated in multiple fields (*n* = 4), with each field containing at least 20 cells. (C) Quantification of bacterial adherence showing the number of recovered bacteria (CFU/well) after plating cell lysates onto LB agar plates supplemented with appropriate antibiotics. Error bars represent means ± SD from six biological replicates. Statistical significance was determined by using one-way ANOVA followed by a *post hoc* Tukey test. *, *P* < 0.05; **, *P* < 0.01; ***, *P* < 0.001; ****, *P* < 0.0001. (D) Time-lapse microscopy of Lifeact::GFP-expressing HeLa cells infected with mCherry-expressing EPEC strains. White arrowheads indicate clusters of pedestal-forming bacteria.

Subsequent analyses of the dynamics of AE lesion formation by these strains showed that infections with BA320 resulted in a higher number of cells with pedestals compared to KOct1- and KOct2-infected cells. No significant difference was observed between KOct1- and KOct2-infected cells (Fig. 4A and B). Notably, EspFu-producing strains were significantly more adherent than their *espFu* isogenic mutants (BA320 versus KO and KOct1 versus KOct2), with BA320 having the highest levels of bacterial attachment (Fig. 4C). Live cell imaging also revealed a higher number of attached bacteria and pedestals formed by BA320 in comparison to KOct1 and KOct2 strains over the course of infection (Fig. 4D, see also Videos S1 to 3 in the supplemental material). Similarly, the infection of HeLa cells with a *tir* mutant complemented with a plasmid expressing Tir_BA320_ (pTir) resulted in a higher number of attached bacteria and cells with pedestals compared to its isogenic pair KOct1 (Fig. S2), indicating that the differences observed in the dynamics of AE lesion formation were more likely due to the mechanism of pedestal formation rather than the location and/or mode of expression of *tir* (i.e., chromosomal in BA320 and plasmid-encoded in KOct1 and KOct2). Collectively, these results indicate that both bacterial attachment and pedestal formation are more efficient in the presence of EspFu, especially in combination with Tir_S_, which is not tyrosine phosphorylated.

10.1128/mBio.00617-20.2 FIG S2 Combination of EspFu and Tir_S_ is associated with increased bacterial attachment and pedestal formation. (A) Immunofluorescence assay showing production and translocation of Tir to the host cell. HeLa cells were infected with the indicated strains, fixed, and then labeled with rabbit anti-Tir polyclonal serum (red), phalloidin-FITC (actin, green), and DAPI (bacteria and cell nuclei, blue). Scale bar, 20 μm. (B) Quantification of FAS showing the percentage of cells with EPEC forming actin pedestals. The number of cells with pedestals was enumerated in multiple fields, with each field containing at least 20 cells. (C) Quantification of bacterial adherence showing the number of recovered bacteria (CFU/well) after plating cell lysates onto LB agar plates supplemented with appropriate antibiotics. Error bars represent means ± the SD from three biological replicates. Statistical significance was determined by using an unpaired Student *t* test. *, *P* < 0.05; **, *P* < 0.01. Download FIG S2, TIF file, 2.5 MB. Copyright © 2020 Martins et al. 2020 Martins et al. This content is distributed under the terms of the Creative Commons Attribution 4.0 International license.

### Actin pedestal formation induced by EPEC activates inflammation-related pathways on epithelial cells.

**FIG 5:**
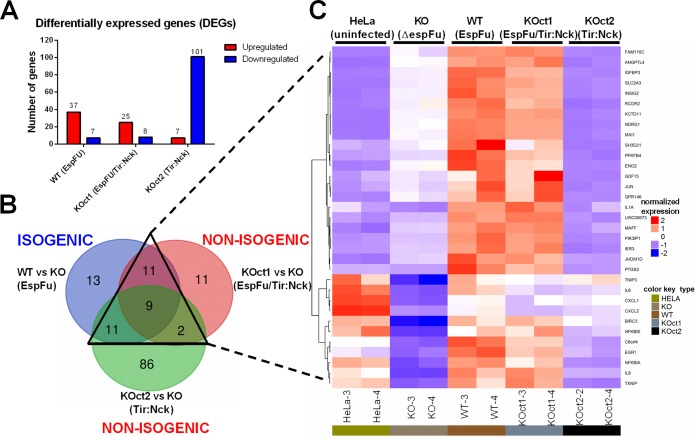
RNA-Seq analysis of the transcriptional response of host epithelial cells to actin polymerization induced by EPEC. (A) Number of up (red) and downregulated (blue) differentially expressed genes (DEGs) in HeLa cells infected with pedestal-forming strains (WT, KOct1, and KOct2) compared to cells infected with an *espFu* mutant (KO). DEGs were identified based on an FDR of ≤0.01 and an FC of −1 ≥ log_2_FC ≥ 1. (B) Venn diagram of overlapping DEG profiles. DEGs shared among the isogenic (WT versus KO) and nonisogenic (KOct1 versus KO and KOct2 versus KO) comparisons are indicated by a triangle with solid lines. (C) Hierarchical clustering and heat map showing the expression pattern of 33 genes that were differentially expressed in response to distinct actin polymerization pathways employed by EPEC.

Transcriptomic analysis has provided valuable insights on bacterial-host interactions, as well on the processes leading to disease, including several studies using pathogenic E. coli infections (36–38). To investigate how different actin assembly pathways affect transcription of host cells, we performed RNA-Seq analysis using total mRNA purified from HeLa monolayers infected with the WT and the engineered BA320 strains for 6 h. This time point was chosen because after 6 h of infection, the effectors have been delivered, and actin has been rearranged into pedestals. The transcriptomic profiles of cells infected with pedestal-forming strains (BA320, KOct1, and KOct2) were compared to that from cells infected with a pedestal-deficient strain (KO). Uninfected cells were used as a control. By applying statistical cutoffs of a false discovery rate (FDR) of ≤0.01 and fold change (FC) cutoffs of −1 ≥ log_2_FC ≥ 1, we found that 44, 33, and 108 genes were differentially expressed in cells infected with BA320, KOct1, and KOct2, respectively (Fig. 5A). It is important to point out that these pairwise comparisons involve both isogenic (WT versus KO) and not isogenic strains (KOct1 versus KO and KOct2 versus KO). Remarkably, cells infected with EspFu-expressing strains showed a greater number of upregulated genes, while the transcriptome of cells infected with a strain that exclusively uses the Tir-Nck pathway had predominantly downregulated genes (Table S2).

Overall, a total of 143 genes were differentially expressed in cells infected with pedestal-forming strains compared to KO infections. With a few exceptions, the magnitude of expression of these genes was considerably small (a fold change of <4) (Table S2). Then, we restricted our analysis to only 33 differentially expressed genes (DEGs) that were shared among isogenic and nonisogenic comparisons in order to identify genes potentially modulated in response to pedestal formation induced by EPEC (Fig. 5B). Interestingly, hierarchical clustering of these 33 genes showed a very similar expression pattern in cells infected with EspFu-expressing strains (WT and KOct1), indicating that this could constitute a common set of genes modulated in response to EspFu-mediated actin assembly pathways (Fig. 5C).

**FIG 6:**
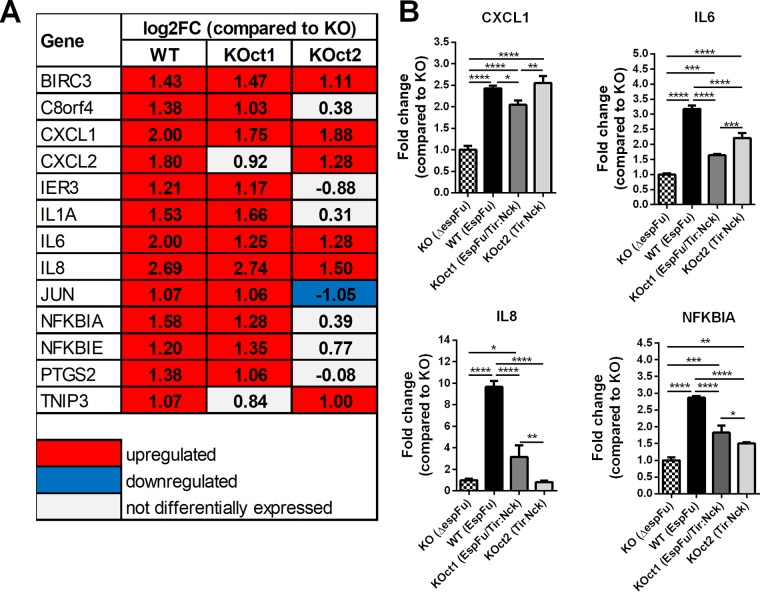
Actin pedestal formation by EPEC induces the expression of genes involved in inflammation and apoptosis. (A) Expression levels of proinflammatory and/or apoptotic genes differentially expressed in cells infected with pedestal-forming strains compared to cells infected with the *espFu* mutant. The values (log_2_FC) shown were obtained from the RNA-Seq data set. (B) Validation of RNA-Seq data by qRT-PCR assay, showing the expression levels of CXCL1, IL-6, IL-8, and NFKBIA genes. Data were normalized to B2M (endogenous control) and presented as means ± the SD from four biological replicates. Statistical significance was determined by using one-way ANOVA, followed by a *post hoc* Tukey test. *, *P* < 0.05; **, *P* < 0.01; ***, *P* < 0.001; ****, *P* < 0.0001.

Among the genes commonly modulated in response to pedestal formation, many were involved with inflammatory and/or apoptotic processes, particularly those that encode proinflammatory cytokines (IL-1A, IL-6, and IL-8), chemokines (CXCL1 and CXCL2), antiapoptotic factors (BIRC3), and prostaglandins (PTGS2), as well as genes that limit the immune response (NFKBIA, NFKBIE, and TNIP3), among others (Fig. 6A). Some of these genes were preferentially regulated in response to the EspFu-mediated pedestal formation, such as C8orf4, IER3, IL1A, NFKBIA, NFKBIE, and PTGS2. Validation of the transcriptome data by quantitative real-time PCR showed that the relative expression levels of four tested genes (CXCL1, IL-6, IL-8, and NFKBIA) exhibited the same regulatory trends as compared with RNA-Seq data set, except for IL-8 in KOct2-infected cells that did not show a significant change in comparison with cells infected with KO (Fig. 6B).

We then hypothesized that the stronger inflammatory response triggered by EspFu-expressing strains could be most likely due to the presence of a greater number of AE lesions and attached bacteria, leading to a higher exposure to surface antigens such as LPS, flagellin, and immunogenic components of the T3SS, which are known to activate the inflammatory response in infections by AE pathogens (39–42). To test this, we analyzed the expression profile of some proinflammatory genes in conditions where the bacterial loads on the surface of cells were normalized. By increasing the multiplicity of infection (MOI) from 10 to 100, we observed that KO and KOct2 adhered at the same levels than their isogenic pairs BA320 (Fig. S3C) and KOct1 (Fig. S3G), respectively. As expected, the enhanced bacterial attachment by KO strain did not result in a significant increase of the number of cells with pedestals (Fig. S3A and B), whereas KOct2 formed pedestals more efficiently under these conditions (Fig. S3E and F). However, cells infected with BA320 showed a higher expression level of CXCL1 and IL-8 genes than cells infected with a similar bacterial load of KO (Fig. S3D), suggesting that the upregulation of these proinflammatory genes was mostly due to the pedestal formation driven by EspFu. Similarly, the infection of cells with KOct1, that employs EspFu in addition to Tir:Nck-mediated actin polymerization pathway, resulted in a higher expression of these same genes compared to cells showing the same bacterial load of KOct2 (Fig. S3H). Taken together, these results suggest that the activation of proinflammatory genes could be more likely due to the presence of an EspFu-mediated actin assembly mechanism rather than the enhanced exposure to bacterial surface antigens such as LPS, flagellin, and T3SS.

10.1128/mBio.00617-20.3 FIG S3 Activation of proinflammatory genes by EPEC depends on the mechanism of pedestal formation rather than an enhanced bacterial association with the epithelium. (A and E) FAS assay on HeLa cells infected with BA320 (WT, MOI of 10), KOct1 (MOI of 10), KOct2 (MOI of 100), and KO (MOI of 100) strains. Scale bar, 20 μm. (B and F) Quantification of FAS showing the percentage of cells with EPEC-forming actin pedestals. (C and G) Quantification of bacterial adherence showing the number of recovered bacteria (CFU/well) after plating cell lysates onto LB agar plates supplemented with appropriate antibiotics. Error bars represent means ± the SD from six biological replicates. (D and H) qRT-PCR analysis of the expression levels of CXCL1 and IL8 genes in HeLa cells infected with similar bacterial loads of EPEC strains. Data were normalized to B2M (endogenous control) and presented as means ± the SD from three biological replicates. Statistical significance was determined by using an unpaired Student *t* test. *, *P* < 0.05; **, *P* < 0.01; ***, *P* < 0.0001; ns, not significant. Download FIG S3, TIF file, 2.9 MB. Copyright © 2020 Martins et al. 2020 Martins et al. This content is distributed under the terms of the Creative Commons Attribution 4.0 International license.

**FIG 7:**
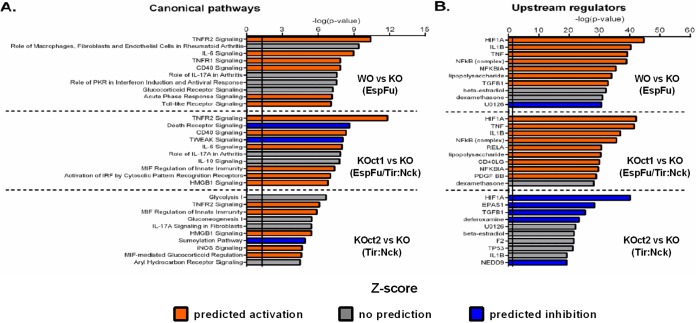
Top canonical signaling pathways and upstream regulators modulated in response to the different mechanisms of pedestal formation employed by EPEC. Ingenuity Pathway Analysis (IPA) showing the 10 most significantly affected canonical pathways (A) and upstream regulators (B) in HeLa cells infected with pedestal-forming EPEC strains. The significance score (negative log of the *P* value) is indicated by the bars on the *x* axis, and the solid vertical black lines represent a *P* value significance threshold of 0.05. The color scheme depicted is based on Z-scores: orange indicates predicted activation, blue indicates predicted inhibition, and gray indicates that certain canonical pathway or upstream regulator is predicted to have significant involvement but the activation state cannot be determined based on the gene expression data (undetermined directionality).

We next used the Ingenuity Pathway Analysis (IPA) tool to investigate possible biological interactions between the DEGs and to identify functional networks (canonical signaling pathways and upstream regulators) associated with the different actin assembly pathways induced by EPEC. To maximize the expression data available to the IPA software, we loosened the inclusion criteria by applying filters of −0.5 ≥ log_2_FC ≥ 0.5 and FDR ≤ 0.05. Figure 7A shows the 10 most significantly affected canonical pathways in each comparison, ranked according to the *P* value, as well as the activation status based on the Z-score. Notably, most of these pathways were related to infectious processes, inflammatory responses, and immunological diseases, reinforcing our hypothesis that AE lesions induced by EPEC could activate inflammatory responses in host epithelial cells. Some pathways were commonly regulated in response to all pedestal-forming strains, notably TNFR2 signaling (Fig. S4A), whereas others, such as IL-6 signaling (Fig. S4B) and MIF-mediated regulation of innate immunity, were preferentially activated in response to EspFu- or Tir:Nck-dependent mechanisms, respectively. We did not observe any pathway related to actin cytoskeleton signaling among those that were most significantly affected, suggesting that the expression of actin-related genes was not changed at this time point (6 h of infection), when the pedestals are already formed. Although we have not tested this, we speculate that the actin cytoskeleton signaling can be affected in the early stages of the infection, when the actin polymerization is triggered.

10.1128/mBio.00617-20.4 FIG S4 IPA analysis of TNF receptor 2 (TNFR2; A) and interleukin-6 (IL-6; B) signaling pathways activated in cells infected with pedestal-forming EPEC strains. Genes that showed differential expression are highlighted in color. Color intensity reflects magnitude of change (red, upregulated; green, downregulated). Genes without color were not affected by the treatment. Solid lines represent direct interactions. Download FIG S4, TIF file, 1.2 MB. Copyright © 2020 Martins et al. 2020 Martins et al. This content is distributed under the terms of the Creative Commons Attribution 4.0 International license.

The prediction of transcription factors (upstream regulators) potentially related to the gene expression changes reported in our data were also performed using IPA tool. Figure 7B shows the 10 most significantly affected upstream regulators in each comparison based on the *P* and *Z*-score values. Notably, most of these regulators were predicted to be activated in infections with EspFu-expressing strains and inhibited in cells infected with KOct2 strain. HIF1A was predicted to be the most relevant affected upstream regulator in all infections (Fig. S5A). Genes encoding proinflammatory cytokines, such as IL-1B and tumor necrosis factor (TNF) (Fig. S5B), were also found among the most affected upstream regulators, mainly in response to EspFu-mediated actin assembly.

10.1128/mBio.00617-20.5 FIG S5 Molecular networks of hypoxia-induced factor 1α (HIF1A; A), interleukin-1β (IL1B; B), and tumor necrosis factor (TNF; B) identified by IPA in cells infected with pedestal-forming EPEC strains. Genes that showed differential expression are highlighted in color. Color intensity reflects magnitude of change (red, upregulated; green, downregulated). Genes without color were not affected by the treatment. Solid lines represent direct interactions and dashed lines indirect interactions. Download FIG S5, TIF file, 2.1 MB. Copyright © 2020 Martins et al. 2020 Martins et al. This content is distributed under the terms of the Creative Commons Attribution 4.0 International license.

### Predominance of an anti-inflammatory response in epithelial cells infected with a pedestal-deficient EPEC strain.

**FIG 8:**
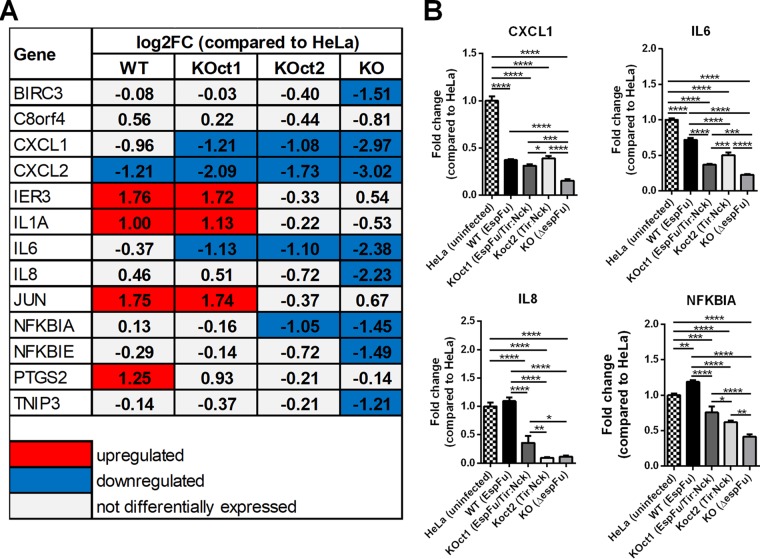
EPEC suppression of the inflammatory response is evidenced in the absence of a robust pedestal formation. (A) Expression levels of proinflammatory and/or apoptotic genes which were differentially expressed in cells infected with pedestal-forming (WT, KOct1 and KOct2) or a pedestal-deficient strain compared to uninfected HeLa cells. The values (log_2_FC) shown were obtained from the RNA-Seq data set. (B) Validation of RNA-Seq data by qRT-PCR assay, showing the expression levels of CXCL1, IL-6, IL-8, and NFKBIA genes. Data were normalized to B2M (endogenous control) and are presented as means ± the SD from four biological replicates. Statistical significance was determined by using one-way ANOVA, followed by a *post hoc* Tukey test. *, *P* < 0.05; **, *P* < 0.01; ***, *P* < 0.001; ****, *P* < 0.0001.

Interestingly, many immune-related genes that were upregulated in our data set were actually strongly downregulated in cells infected with a pedestal-deficient strain (KO) (Fig. 5C and 8), suggesting that EPEC can also induce an anti-inflammatory effect in epithelial cells in the absence of a robust pedestal formation. Thus, EPEC strains that generate pedestals, especially through EspFu mechanisms, are able to reverse this effect, which explains the fact that infections with pedestal-forming strains (BA320, KOct1, and KOct2) did not significantly affected the expression of proinflammatory genes compared to uninfected HeLa cells (mock control) (Fig. 5C and 8).

**FIG 9:**
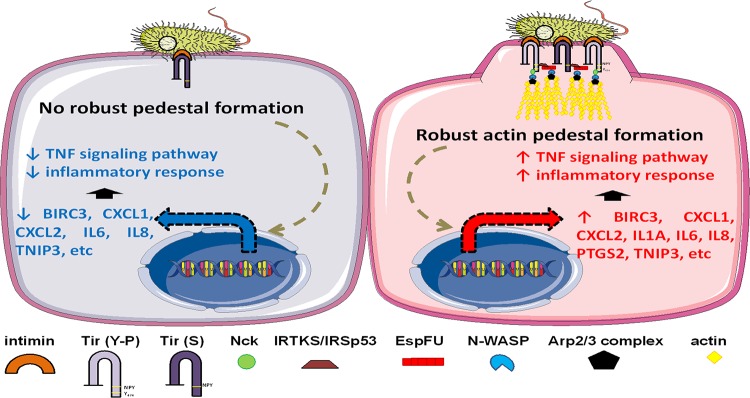
Proposed model for the interaction of EPEC with the host epithelial cell. The formation of actin pedestals, especially when induced by EspFu-dependent mechanisms, activates inflammatory signaling pathways in the host cell. In the absence of a robust pedestal formation, most proinflammatory genes are downregulated, possibly due to the activity of translocated T3SS effectors which dampen the inflammatory response. Thus, the EPEC-induced inflammation is a balance between pro- and anti-inflammatory events.

Collectively, our data demonstrate that genes and pathways involved in inflammation are activated in response to pedestal formation induced by EPEC, especially when mediated by EspFu. Moreover, we also show that EPEC can employ anti-inflammatory mechanisms, as evidenced by the significant downregulation of these proinflammatory genes in epithelial cells infected with a pedestal-deficient strain (Fig. 9).

## DISCUSSION

The hallmark of EPEC pathogenesis is the ability to utilize effector proteins to manipulate host cellular processes, leading to intimate bacterial attachment and intestinal colonization (43). However, several facets of these effectors remain unexplored. A large number of EPEC strains, prominently from the O55:H7 serotype, carry the *espFu* gene (16, 18), indicating that EspFu is important for the pathogenesis of this pathotype. This distribution of *espFu* is interesting, given that EHEC O157:H7, which relies on EspFu for pedestal formation (12), evolved from EPEC O55:H7 (31). Here, we show that in addition for the requirement of EspFu for bacterial attachment and pedestal formation, this effector also activates an inflammatory response on epithelial cells.

Previous studies demonstrated that isogenic *espFu* mutants of EPEC (44) and EHEC (45) were less adherent and did not induce robust actin rearrangement on epithelial cells. The introduction of an EspFu-expressing plasmid into a nonadherent strain of EPEC resulted in adherence and pedestal formation on HeLa cells (30). EspFu also increased the adherence of EPEC O125:H6 to human intestinal biopsy specimens *in vitro* (46) and enhanced the intestinal colonization by EHEC in infant rabbits and gnotobiotic piglets (47). Therefore, it has been proposed that the EspFu-driven pedestal could facilitate actin-mediated movement through the epithelium, enabling transmission to neighboring cells, thus promoting bacterial expansion beyond the original sites of infection and improving epithelial colonization (48).

AE lesion formation is a dynamic process that requires the timely and coordinate expression of the bacterial virulence machinery (49). AE pathogens evolved complex mechanisms to trigger actin polymerization on host cells (50). Here, we show that EspFu-mediated actin polymerization promoted a higher bacterial attachment and pedestal formation in comparison to the Tir:Nck-dependent pathway (Fig. 3). This higher efficiency is probably due to the presence of multiple PRRs domains in EspFu, which allows this effector to bind to more N-WASP molecules than Nck, which has only three SH3 domains (51, 52). Surprisingly, the combination of these two mechanisms (EspFu and Tir-phosphorylation) did not increase pedestal formation (Fig. 3 and Fig. S2), suggesting that EspFu-driven actin polymerization is more efficient in association with the nonphosphorylated form of Tir (Tir_S_). Our results corroborate previous findings showing that EspFu-expressing EPEC can more efficiently colonize epithelial cells in the presence of Tir_EHEC_ (nonphosphorylated) compared to Tir_EPEC_ (phosphorylated) (48). In addition, one can speculate that the host proteins IRTKS and Nck can compete for binding to Tir due to the proximity of their binding motifs. Thus, Tir-IRTKS binding would be favored in the absence of tyrosine phosphorylation, leading to the recruitment of EspFu and triggering the downstream signaling required for actin polymerization.

The analyses of the transcriptional response of host epithelial cells to AE lesion formation (Fig. 5 and 8) also demonstrates that many genes involved in immune processes (i.e., inflammation and apoptosis) were differentially expressed in cells infected with pedestal-forming EPEC strains, most prominently in response to EspFu-mediated actin polymerization. Activation of pathways and transcription factors associated with inflammation and apoptosis on epithelial cells required AE lesion formation (Fig. 5 and 8). Although it is tempting to speculate that the stronger inflammatory response triggered by EspFu-expressing strains was most likely due to an enhanced bacterial association with the epithelium, our data suggest an additional mechanism by which EspFu-mediated actin polymerization can activate inflammation, since cells infected with similar bacterial loads responded differentially to the distinct actin polymerization mechanisms (Fig. S3). However, further studies are still need to better understand this mechanism.

Remarkably, EPEC can also suppress the inflammatory response, evidenced by the strong repression of proinflammatory genes in cells infected with a pedestal-deficient strain (*espFu* mutant) (Fig. 5 and 8). Previous studies have demonstrated that NF-κB activation is decreased during the course of infection by AE pathogens (53–56) and that this anti-inflammatory activity depends on a functional T3SS (57). In agreement with this, our *in silico* analysis identified many effector-encoding genes in the BA320 genome that could be associated with immunomodulation, such as NleB, NleC, NleD, NleE, and NleH. Interestingly, this immunomodulatory activity was more efficiently counterbalanced by the inflammatory response induced by EspFu-mediated actin polymerization. Thus, an interesting future line of research will be to analyze the kinetics of expression and translocation of effectors involved with cytoskeleton rearrangement (such as Tir and EspFu) and immunomodulation during the infection by EPEC strains that employ different actin polymerization mechanisms.

One can speculate that some important observations of this study could be actually due to pairwise comparisons involving strains that are not isogenic (e.g., WT versus KOct1 and KOct2 versus KO). These strains differ regarding location and mode of expression of *tir*, which could affect the dynamics of infection and, as a consequence, the host response to this process. In fact, it has been shown that the relocation of *tir* to a plasmid can affect its function and only partially restore the phenotype conferred by this effector (58, 59). However, we showed that the expression of Tir_BA320_ from a plasmid restored the ability of a *tir* deletion mutant to form actin pedestals to a level similar to that seen in cells infected with WT strain (Fig. 4 and Fig. S2). Thus, our data suggest that the differences observed in this study, including host response to pedestal formation, were more likely due to the different mechanisms of actin assembly triggered rather than the nonisogenic nature of the strains.

In summary, this study links actin-rearrangement through AE lesion formation with modulation of the host immune responses. It also highlights that different actin-polymerization-mediated pathways differentially impact this immunomodulation. The different distribution and combination of Tir and EspFu alleles in EPEC and EHEC pathotypes contributes to the plasticity of these enteric infections.

## MATERIALS AND METHODS

### Bacterial strains, plasmids, and growth conditions.

The bacterial strains and plasmids utilized in this study are listed in Table S1 in the supplemental material. All strains were routinely grown in Luria-Bertani medium (LB) or low-glucose Dulbecco modified Eagle medium (DMEM) at 37°C. When adequate, media were supplemented with ampicillin (100 μg/ml), kanamycin (50 μg/ml), chloramphenicol (25 μg/ml), or streptomycin (100 μg/ml). Bacterial stocks were kept on LB supplemented with glycerol 20% (vol/vol) at –80°C.

10.1128/mBio.00617-20.6 TABLE S1 Bacterial strains, plasmids, and oligonucleotide primers used in this study. Download Table S1, DOCX file, 0.03 MB. Copyright © 2020 Martins et al. 2020 Martins et al. This content is distributed under the terms of the Creative Commons Attribution 4.0 International license.

10.1128/mBio.00617-20.7 TABLE S2 List of differentially expressed genes (DEGs) in each comparison. Download Table S2, DOCX file, 0.05 MB. Copyright © 2020 Martins et al. 2020 Martins et al. This content is distributed under the terms of the Creative Commons Attribution 4.0 International license.

Nonpolar mutants of *espFu* and *tir* in EPEC BA320 (serotype O55:H7) were constructed by using λ-Red recombination method (60). Briefly, PCR products were amplified from plasmids pKD3 and pKD4 with flanking regions matching *espFu* and *tir*, respectively, and were transformed into BA320 expressing the recombinases from plasmid pKD46. Colonies were selected from chloramphenicol or kanamycin LB plates, and the resistance cassette was resolved using flippase from temperature-sensitive plasmid pCP20, which was then cured through growth at 42°C. The nonpolar mutants were then confirmed by sequencing.

For complementations, *espFu* and *tir* genes were amplified from BA320 and E2348/69 (EPEC O127:H6) genomic DNAs, respectively, using the primers described in Table S1, and then cloned into pACYC184 under the control of the Tet promoter.

### Whole-genome sequencing, assembly, and annotation.

A bacterial culture was grown at 37°C overnight and then centrifuged, and the pellet was used for DNA extraction. Total DNA was sequenced in a Hiseq1500 (Illumina-USA) using the Rapid protocol to obtain 2 × 250 paired-end reads, according to the manufacturer´s recommendations. Raw data were processed (Trimmomatic version 0.38) (61) and genome *de novo* assembly was carried out using a conciliation tool (CISA version 1.3) (62) after four independent *de novo* assemblies, using different assemblers (ABySS version 2.1.0, VelvetOptimizer version 2.2.6, SOAPdenovo version 2.01, and SPAdes version 3.13.0) (63–66). Plasmid *de novo* assembly was done using Recycler (version 0.7) (67), and only assembled plasmids that were also detected by PlasmidSeeker (version 1.0) (68) on total genome assembly were considered valid plasmid assemblies. Genome and plasmid annotations were carried out using Prokka (version 1.14.3) (69).

### *In silico* analyses.

The distribution of T3SS effectors in the BA320 genome was determined by BLAST score ratio (BSR) analysis (70) using TBLASTN under default parameters, as previously described (71). The T3SS effector protein sequences used as query sequences were obtained from genomes of EHEC O157:H7 Sakai (72), EPEC O127:H6 E2348/69 (73, 74), and EPEC O111:H– B171 (75). Prophages were identified using PHASTER (76). Multiple sequence alignments and analysis were performed using Geneious (Biomatters), EasyFig (77), and Clustal Omega (78).

### Growth curve.

Overnight cultures in LB were diluted 1:1,000 in 3 ml of low-glucose DMEM on a 6-well plate and then statically grown for 6 h at 37°C under 5% CO_2_. Aliquots (10 μl) were collected every 1.5 h, serially diluted in phosphate-buffered saline (PBS), and plated onto LB agar containing selective antibiotics for CFU enumeration. All strains were tested in triplicates, and at least two independent experiments were performed.

### Western blotting for secreted proteins.

Secreted proteins from cultures grown for 6 h in low-glucose DMEM at 37°C and 5% CO_2_ were prepared as previously described (7). Portions (10 μg) of bovine serum albumin (BSA) were added to secreted protein samples as a loading control. The proteins were separated by SDS-PAGE, transferred to polyvinylidene fluoride (polyvinylidene difluoride) membranes, probed with rabbit polyclonal anti-EspB serum, and visualized with enhanced chemiluminescence (Thermo Fisher) using the ChemiDoc touch imaging system (software 1.0.0.15) with Image Lab 5.2.1 software for image capture display.

### Cell culture and bacterial infections.

HeLa (human cervical adenocarcinoma) cells were maintained in high-glucose DMEM supplemented with 10% fetal bovine serum (FBS) and penicillin-streptomycin-glutamine and grown at 37°C and 5% CO_2_. The Lifeact::GFP-expressing HeLa cell line was obtained with the Flip-In system (Invitrogen), according to the method of Gruber and Sperandio (49) and maintained in high-glucose DMEM supplemented with 10% FBS, penicillin-streptomycin-glutamine, and hygromycin (50 μg/ml). Hygromycin was not added when cells were split before infection.

For infection assays, cells grown overnight to about 80% confluence at 37°C and 5% CO_2_ were washed with PBS, kept in fresh low-glucose DMEM supplemented with 10% FBS, and then infected at an MOI of 10, unless otherwise stated, with overnight bacterial cultures statically grown in LB. Infections were allowed to proceed for 6 h at 37°C and 5% CO_2_, being the medium replaced after 3 h. Inoculum inputs were verified by plating onto LB plates containing selective antibiotics.

### FAS assay.

Fluorescent actin staining (FAS) assays were performed as described by Knutton et al. (79) to examine pedestal formation. HeLa cells were cultivated in 12-well plates with rounded glass coverslips. After 6 h of infection, the cells were washed with PBS, fixed with formaldehyde and permeabilized with 0.2% Triton X-100. The preparations were then treated with fluorescein isothiocyanate (FITC)-labeled phalloidin and 4′,6′-diamidino-2-phenylindole (DAPI) to visualize actin accumulation and bacteria/HeLa DNA, respectively. Slides were mounted with ProLong antifade (Molecular Probes) and visualized with a Zeiss LSM880 confocal laser scanning microscope. Pedestal formation was quantified by randomly imaging different fields of view while recording the number of cells showing actin accumulation foci. At least four fields were enumerated for each condition, with each field containing at least 20 cells. All strains were tested in triplicates and at least three independent experiments were performed.

### Live-cell imaging.

Lifeact::GFP-expressing HeLa cells were cultured in 35-mm glass-bottom dishes (MatTek) and then infected with mCherry-expressing bacteria (stained in red) at an MOI of 100. Infections were allowed to continue for 2 h at 37°C and 5% CO_2_; the cells were then washed and visualized by live-cell imaging with an Olympus Fluoview Fv10i confocal laser scanning microscope. Images were taken every 6 min for 2 h.

### Quantitative adherence assay.

HeLa cells were seeded in 24-well plates, infected for 6 h as described above and then extensively washed with PBS to remove the nonadherent bacteria. Cells were lysed in PBS plus 1% Triton X-100 for 30 min with rocking at room temperature, and recovered bacteria were then serially diluted and plated onto LB plates containing selective antibiotics. Colonies were enumerated after overnight incubation at 37°C. All strains were tested in triplicates and at least three independent experiments were performed.

### SEM.

Infection assays were performed in 24-well plates with rounded glass coverslips for 6 h as described above and then processed according to the method of Knutton (80) to visualize the bacterial interaction with the cell surface. Briefly, preparations were fixed with a solution of 4% paraformaldehyde and 3% glutaraldehyde in 0.1 M sodium cacodylate buffer (pH 7.2), postfixed with 1% osmium tetroxide, and dehydrated with ethanol (60, 70, 80, 90, 95, and 100%). Samples were then subjected to critical point drying with carbon dioxide, mounted on stubs, and coated with a thin layer of gold prior to the visualization under scanning electron microscopy (SEM; QUANTA 250; FEI Company) operating at 12.5 kV and a working distance of 6.6 mm.

### Immunofluorescence assay.

HeLa cells were cultured in 24-well plates with rounded glass coverslips or 8-well Lab-Tek chamber slides and then infected for 6 h at 37°C and 5% CO_2_. After the infections, the cells were washed with PBS, fixed with formaldehyde, permeabilized with PBS plus 0.2% Triton X-100, and blocked with PBS plus 2% BSA. Samples were then probed with rabbit polyclonal anti-Tir (1:2,000) primary antibodies and Alexa Fluor 566-goat anti-rabbit (Molecular Probes) secondary antibody at a dilution of 1:1,000. Actin and DNA were stained with FITC-phalloidin (1:50) and DAPI (1:2,000), respectively. To detect the phosphorylation of Tir, the preparations were treated with monoclonal anti-phosphotyrosine clone PY20 antibody conjugated with FITC (Thermo Fisher) at a dilution of 1:100, and DNA was stained with propidium iodide (1:1,000). Slides were mounted with ProLong antifade and visualized with a Zeiss LSM880 confocal laser scanning microscope.

### RNA sequencing library preparation and data analysis.

For transcriptomic analysis, HeLa cells were grown in 6-well plates and infected for 6 h at 37°C and 5% CO_2_. Following the incubation period, both infected and uninfected cells were washed with PBS, and total RNA was isolated from biological replicates with an RNeasy minikit (Qiagen) according to the manufacturer’s instructions. RNA samples were treated with DNase I (Thermo Fisher) and repurified using RNeasy minikit columns.

A total of 10 μg from each DNase-treated RNA sample was processed for sequencing. Briefly, RNA quality and integrity were analyzed on the Agilent 2100 BioAnalyzer (Agilent Technologies). Samples with an RNA Integrity Number (RIN) of >8 were subjected to an enrichment of polyadenylated RNA, which was used in the preparation of cDNA libraries with TruSeq RNA Sample Prep (Illumina), according to the manufacturer’s instructions. cDNA was A-tailed, and indexed adapters were ligated. After adapter ligation, samples were PCR amplified and purified with AMPure XP beads and then validated again on the Agilent 2100 Bioanalyzer. Samples were quantified with the Qubit fluorometer before being normalized, pooled, and then sequenced on the Illumina NextSeq 500 with read configuration as 75-bp, single-end reads. Totals of 25 to 40 million reads were generated for each sample. Two biological replicates for each condition were tested.

The Fastq files were subjected to quality check using fastqc (version 0.11.2, http://www.bioinformatics.babraham.ac.uk/projects/fastqc) and fastq_screen (version 0.4.4, http://www.bioinformatics.babraham.ac.uk/projects/fastq_screen) and trimmed using fastq-mcf (ea-utils/version 1.1.2-806, https://github.com/ExpressionAnalysis/ea-utils). Trimmed fastq files were mapped to Homo sapiens reference genome (hg19, UCSC version from igenomes) using Tophat (81). Duplicates were marked using picard-tools (version 1.127, https://broadinstitute.github.io/picard/). Read counts were generated using featureCounts (82), and the differential expression analysis was performed using edgeR (83). For differential expression analysis, statistical cutoffs with an FDR of ≤0.01 and FC cutoffs of −1 ≥ log_2_FC ≥ 1 were used to identify statistically significant and possibly biologically relevant differentially regulated transcripts. Pathway and network analysis were performed using Qiagen’s IPA tool. Heat maps were generated using ComplexHeatmap (84) package.

### Quantitative real-time PCR assays.

For RNA-Seq validation, HeLa cells were infected, and RNA samples were isolated with TRIzol reagent (Thermo Fisher) according to the manufacturer’s instructions. A total of 1 μg of DNase-treated RNA was reverse transcribed to cDNA by using a SuperScript III kit (Thermo Fisher) with random primers. The diluted cDNAs were mixed with validated primers (Table S2) and SYBR green mix, and the reactions were then cycled in 384-well plates on the QuantStudio 6 Flex system (Applied Biosystems). Data were collected using QuantStudio real-time PCR software (Applied Biosystems). All data were normalized to the levels of the beta-2 microglobulin (B2M) gene and then analyzed using the comparative cycle threshold method (ΔΔ*C_T_*).

### Statistical analysis.

Statistical significance was determined by unpaired Student *t* test or one-way analysis of variance (ANOVA [for multiple comparisons]). A *P* value of <0.05 was considered significant.

### Data availability.

This whole-genome shotgun project has been deposited at DDBJ/ENA/GenBank under accession no. NZ_SGUH00000000. The RNA-Seq data can be accessed using accession no. GSE141446 at the NCBI GEO database.

10.1128/mBio.00617-20.8 VIDEO S1 Live-cell imaging of HeLa cells infected with WT (EspFu) strain. Download Movie S1, AVI file, 13.9 MB. Copyright © 2020 Martins et al. 2020 Martins et al. This content is distributed under the terms of the Creative Commons Attribution 4.0 International license.

10.1128/mBio.00617-20.9 VIDEO S2 Live-cell imaging of HeLa cells infected with KOct1 (EspFu/Tir-Nck) strain. Download Movie S2, AVI file, 13.9 MB. Copyright © 2020 Martins et al. 2020 Martins et al. This content is distributed under the terms of the Creative Commons Attribution 4.0 International license.

10.1128/mBio.00617-20.10 VIDEO S3 Live-cell imaging of HeLa cells infected with KOct2 (Tir-Nck) strain. Download Movie S3, AVI file, 13.9 MB. Copyright © 2020 Martins et al. 2020 Martins et al. This content is distributed under the terms of the Creative Commons Attribution 4.0 International license.

## References

[1] Dean P, Kenny B 2009 The effector repertoire of enteropathogenic *Escherichia coli*: ganging up on the host cell. Curr Opin Microbiol 12:101–109. doi:10.1016/j.mib.2008.11.006. 19144561 PMC2697328

[2] Hicks SW, Galan JE 2013 Exploitation of eukaryotic subcellular targeting mechanisms by bacterial effectors. Nat Rev Microbiol 11:316–326. doi:10.1038/nrmicro3009. 23588250 PMC3859125

[3] Hernandes RT, Elias WP, Vieira MA, Gomes TA 2009 An overview of atypical enteropathogenic *Escherichia coli*. FEMS Microbiol Lett 297:137–149. doi:10.1111/j.1574-6968.2009.01664.x. 19527295

[4] Hu J, Torres AG 2015 Enteropathogenic *Escherichia coli*: foe or innocent bystander? Clin Microbiol Infect 21:729–734. doi:10.1016/j.cmi.2015.01.015. 25726041 PMC4497942

[5] Moon HW, Whipp SC, Argenzio RA, Levine MM, Giannella RA 1983 Attaching and effacing activities of rabbit and human enteropathogenic *Escherichia coli* in pig and rabbit intestines. Infect Immun 41:1340–1351. doi:10.1128/IAI.41.3.1340-1351.1983. 6350186 PMC264644

[6] McDaniel TK, Jarvis KG, Donnenberg MS, Kaper JB 1995 A genetic locus of enterocyte effacement conserved among diverse enterobacterial pathogens. Proc Natl Acad Sci U S A 92:1664–1668. doi:10.1073/pnas.92.5.1664. 7878036 PMC42580

[7] Jarvis KG, Giron JA, Jerse AE, McDaniel TK, Donnenberg MS, Kaper JB 1995 Enteropathogenic *Escherichia coli* contains a putative type III secretion system necessary for the export of proteins involved in attaching and effacing lesion formation. Proc Natl Acad Sci U S A 92:7996–8000. doi:10.1073/pnas.92.17.7996. 7644527 PMC41273

[8] Elliott SJ, Wainwright LA, McDaniel TK, Jarvis KG, Deng YK, Lai LC, McNamara BP, Donnenberg MS, Kaper JB 1998 The complete sequence of the locus of enterocyte effacement (LEE) from enteropathogenic *Escherichia coli* E2348/69. Mol Microbiol 28:1–4. doi:10.1046/j.1365-2958.1998.00783.x. 9593291

[9] Kenny B, DeVinney R, Stein M, Reinscheid DJ, Frey EA, Finlay BB 1997 Enteropathogenic *Escherichia coli* (EPEC) transfers its receptor for intimate adherence into mammalian cells. Cell 91:511–520. doi:10.1016/s0092-8674(00)80437-7. 9390560

[10] Kenny B 1999 Phosphorylation of tyrosine 474 of the enteropathogenic *Escherichia coli* (EPEC) Tir receptor molecule is essential for actin nucleating activity and is preceded by additional host modifications. Mol Microbiol 31:1229–1241. doi:10.1046/j.1365-2958.1999.01265.x. 10096089

[11] Campellone KG, Giese A, Tipper DJ, Leong JM 2002 A tyrosine-phosphorylated 12-amino-acid sequence of enteropathogenic *Escherichia coli* Tir binds the host adaptor protein Nck and is required for Nck localization to actin pedestals. Mol Microbiol 43:1227–1241. doi:10.1046/j.1365-2958.2002.02817.x. 11918809

[12] Campellone KG, Robbins D, Leong JM 2004 EspFU is a translocated EHEC effector that interacts with Tir and N-WASP and promotes Nck-independent actin assembly. Dev Cell 7:217–228. doi:10.1016/j.devcel.2004.07.004. 15296718

[13] Garmendia J, Phillips AD, Carlier MF, Chong Y, Schuller S, Marches O, Dahan S, Oswald E, Shaw RK, Knutton S, Frankel G 2004 TccP is an enterohaemorrhagic *Escherichia coli* O157:H7 type III effector protein that couples Tir to the actin-cytoskeleton. Cell Microbiol 6:1167–1183. doi:10.1111/j.1462-5822.2004.00459.x. 15527496

[14] de Groot JC, Schlüter K, Carius Y, Quedenau C, Vingadassalom D, Faix J, Weiss SM, Reichelt J, Standfuss-Gabisch C, Lesser CF, Leong JM, Heinz DW, Büssow K, Stradal TEB 2011 Structural basis for complex formation between human IRSp53 and the translocated intimin receptor Tir of enterohemorrhagic *Escherichia coli*. Structure 19:1294–1306. doi:10.1016/j.str.2011.06.015. 21893288 PMC4063679

[15] Vingadassalom D, Kazlauskas A, Skehan B, Cheng HC, Magoun L, Robbins D, Rosen MK, Saksela K, Leong JM 2009 Insulin receptor tyrosine kinase substrate links the *Escherichia coli* O157:H7 actin assembly effectors Tir and EspF(U) during pedestal formation. Proc Natl Acad Sci U S A 106:6754–6759. doi:10.1073/pnas.0809131106. 19366662 PMC2672544

[16] Garmendia J, Ren Z, Tennant S, Midolli Viera MA, Chong Y, Whale A, Azzopardi K, Dahan S, Sircili MP, Franzolin MR, Trabulsi LR, Phillips A, Gomes TA, Xu J, Robins-Browne R, Frankel G 2005 Distribution of tccP in clinical enterohemorrhagic and enteropathogenic *Escherichia coli* isolates. J Clin Microbiol 43:5715–5720. doi:10.1128/JCM.43.11.5715-5720.2005. 16272509 PMC1287796

[17] Ooka T, Vieira MA, Ogura Y, Beutin L, La Ragione R, van Diemen PM, Stevens MP, Aktan I, Cawthraw S, Best A, Hernandes RT, Krause G, Gomes TA, Hayashi T, Frankel G 2007 Characterization of *tccP2* carried by atypical enteropathogenic *Escherichia coli*. FEMS Microbiol Lett 271:126–135. doi:10.1111/j.1574-6968.2007.00707.x. 17403050

[18] Martins FH, Nepomuceno R, Piazza RMF, Elias WP 2017 Phylogenetic distribution of tir-cytoskeleton coupling protein (*tccP* and *tccP2*) genes in atypical enteropathogenic *Escherichia coli*. FEMS Microbiol Lett 364 doi:10.1093/femsle/fnx101. 28505295

[19] Vieira MA, Dias RCB, Dos Santos LF, Rall VLM, Gomes TAT, Hernandes RT 2019 Diversity of strategies used by atypical enteropathogenic *Escherichia coli* to induce attaching and effacing lesion in epithelial cells. J Med Microbiol 68:940–951. doi:10.1099/jmm.0.000998. 31107199

[20] Baruch K, Gur-Arie L, Nadler C, Koby S, Yerushalmi G, Ben-Neriah Y, Yogev O, Shaulian E, Guttman C, Zarivach R, Rosenshine I 2011 Metalloprotease type III effectors that specifically cleave JNK and NF-κB. EMBO J 30:221–231. doi:10.1038/emboj.2010.297. 21113130 PMC3020117

[21] Muhlen S, Ruchaud-Sparagano MH, Kenny B 2011 Proteasome-independent degradation of canonical NFκB complex components by the NleC protein of pathogenic *Escherichia coli*. J Biol Chem 286:5100–5107. doi:10.1074/jbc.M110.172254. 21148319 PMC3037621

[22] Nadler C, Baruch K, Kobi S, Mills E, Haviv G, Farago M, Alkalay I, Bartfeld S, Meyer TF, Ben-Neriah Y, Rosenshine I 2010 The type III secretion effector NleE inhibits NF-κB activation. PLoS Pathog 6:e1000743. doi:10.1371/journal.ppat.1000743. 20126447 PMC2813277

[23] Newton HJ, Pearson JS, Badea L, Kelly M, Lucas M, Holloway G, Wagstaff KM, Dunstone MA, Sloan J, Whisstock JC, Kaper JB, Robins-Browne RM, Jans DA, Frankel G, Phillips AD, Coulson BS, Hartland EL 2010 The type III effectors NleE and NleB from enteropathogenic *Escherichia coli* and OspZ from *Shigella* block nuclear translocation of NF-κ p65. PLoS Pathog 6:e1000898. doi:10.1371/journal.ppat.1000898. 20485572 PMC2869321

[24] Pearson JS, Riedmaier P, Marches O, Frankel G, Hartland EL 2011 A type III effector protease NleC from enteropathogenic *Escherichia coli* targets NF-κB for degradation. Mol Microbiol 80:219–230. doi:10.1111/j.1365-2958.2011.07568.x. 21306441 PMC3178796

[25] Pearson JS, Hartland EL 2014 The inflammatory response during enterohemorrhagic *Escherichia coli* infection. Microbiol Spectr 2:EHEC-0012-2013. 10.1128/microbiolspec.EHEC-0012-2013 26104206

[26] Pearson JS, Giogha C, Wong Fok Lung T, Hartland EL 2016 The genetics of enteropathogenic *Escherichia coli* virulence. Annu Rev Genet 50:493–513. doi:10.1146/annurev-genet-120215-035138. 27893961

[27] Vossenkamper A, Marches O, Fairclough PD, Warnes G, Stagg AJ, Lindsay JO, Evans PC, Luong Le A, Croft NM, Naik S, Frankel G, MacDonald TT 2010 Inhibition of NF-κB signaling in human dendritic cells by the enteropathogenic *Escherichia coli* effector protein NleE. J Immunol 185:4118–4127. doi:10.4049/jimmunol.1000500. 20833837

[28] Royan SV, Jones RM, Koutsouris A, Roxas JL, Falzari K, Weflen AW, Kim A, Bellmeyer A, Turner JR, Neish AS, Rhee KJ, Viswanathan VK, Hecht GA 2010 Enteropathogenic *Escherichia coli* non-LEE encoded effectors NleH1 and NleH2 attenuate NF-κB activation. Mol Microbiol 78:1232–1245. doi:10.1111/j.1365-2958.2010.07400.x. 21091507 PMC3325542

[29] Bueris V, Sircili MP, Taddei CR, dos Santos MF, Franzolin MR, Martinez MB, Ferrer SR, Barreto ML, Trabulsi LR 2007 Detection of diarrheagenic *Escherichia coli* from children with and without diarrhea in Salvador, Bahia, Brazil. Mem Inst Oswaldo Cruz 102:839–844. doi:10.1590/s0074-02762007005000116. 17992362

[30] Rocha SP, Abe CM, Sperandio V, Bando SY, Elias WP 2011 Atypical enteropathogenic *Escherichia coli* that contains functional locus of enterocyte effacement genes can be attaching-and-effacing negative in cultured epithelial cells. Infect Immun 79:1833–1841. doi:10.1128/IAI.00693-10. 21343354 PMC3088124

[31] Whittam TS, Reid SD, Selander RK 1998 Mutators and long-term molecular evolution of pathogenic *Escherichia coli* O157:H7. Emerg Infect Dis 4:615–617. doi:10.3201/eid0404.980411. 9866737 PMC2640239

[32] Girón JA, Ho ASY, Schoolnik GK 1991 An inducible bundle-forming pilus of enteropathogenic *Escherichia coli*. Science 254:710–713. doi:10.1126/science.1683004. 1683004

[33] Mellies JL, Barron AMS, Carmona AM 2007 Enteropathogenic and enterohemorrhagic *Escherichia coli* virulence regulation. Infect Immun 75:4199–4210. doi:10.1128/IAI.01927-06. 17576759 PMC1951183

[34] Abe CM, Trabulsi LR, Blanco J, Blanco M, Dahbi G, Blanco JE, Mora A, Franzolin MR, Taddei CR, Martinez MB, Piazza RM, Elias WP 2009 Virulence features of atypical enteropathogenic *Escherichia coli* identified by the *eae*(+) EAF-negative *stx*(–) genetic profile. Diagn Microbiol Infect Dis 64:357–365. doi:10.1016/j.diagmicrobio.2009.03.025. 19442475

[35] Cepeda-Molero M, Berger CN, Walsham ADS, Ellis SJ, Wemyss-Holden S, Schüller S, Frankel G, Fernández LÁ 2017 Attaching and effacing (A/E) lesion formation by enteropathogenic *Escherichia coli* on human intestinal mucosa is dependent on non-LEE effectors. PLoS Pathog 13:e1006706. doi:10.1371/journal.ppat.1006706. 29084270 PMC5685641

[36] He X, Mishchuk DO, Shah J, Weimer BC, Slupsky CM 2013 Cross-talk between *E. coli* strains and a human colorectal adenocarcinoma-derived cell line. Sci Rep 3:3416. doi:10.1038/srep03416. 24301462 PMC3849634

[37] Kieckens E, Rybarczyk J, Li RW, Vanrompay D, Cox E 2016 Potential immunosuppressive effects of *Escherichia coli* O157:H7 experimental infection on the bovine host. BMC Genomics 17:1049. doi:10.1186/s12864-016-3374-y. 28003017 PMC5178093

[38] Yang WE, Suchindran S, Nicholson BP, McClain MT, Burke T, Ginsburg GS, Harro CD, Chakraborty S, Sack DA, Woods CW, Tsalik EL 2016 Transcriptomic analysis of the host response and innate resilience to enterotoxigenic *Escherichia coli* infection in humans. J Infect Dis 213:1495–1504. doi:10.1093/infdis/jiv593. 26787651 PMC5007733

[39] Khan MA, Bouzari S, Ma C, Rosenberger CM, Bergstrom KS, Gibson DL, Steiner TS, Vallance BA 2008 Flagellin-dependent and -independent inflammatory responses following infection by enteropathogenic *Escherichia coli* and *Citrobacter rodentium*. Infect Immun 76:1410–1422. doi:10.1128/IAI.01141-07. 18227166 PMC2292885

[40] Litvak Y, Sharon S, Hyams M, Zhang L, Kobi S, Katsowich N, Dishon S, Nussbaum G, Dong N, Shao F, Rosenshine I 2017 Epithelial cells detect functional type III secretion system of enteropathogenic *Escherichia coli* through a novel NF-κB signaling pathway. PLoS Pathog 13:e1006472. doi:10.1371/journal.ppat.1006472. 28671993 PMC5510907

[41] Sampaio SCF, Gomes TAT, Pichon C, Du Merle L, Guadagnini S, Abe CM, Sampaio JLM, Le Bouguénec C 2009 The flagella of an atypical enteropathogenic *Escherichia coli* strain are required for efficient interaction with and stimulation of interleukin-8 production by enterocytes *in vitro*. Infect Immun 77:4406–4413. doi:10.1128/IAI.00177-09. 19620340 PMC2747955

[42] Schuller S, Lucas M, Kaper JB, Giron JA, Phillips AD 2009 The *ex vivo* response of human intestinal mucosa to enteropathogenic *Escherichia coli* infection. Cell Microbiol 11:521–530. doi:10.1111/j.1462-5822.2008.01275.x. 19134113 PMC2676445

[43] Frankel G, Phillips AD 2008 Attaching effacing *Escherichia coli* and paradigms of Tir-triggered actin polymerization: getting off the pedestal. Cell Microbiol 10:549–556. doi:10.1111/j.1462-5822.2007.01103.x. 18053003

[44] Whale AD, Hernandes RT, Ooka T, Beutin L, Schuller S, Garmendia J, Crowther L, Vieira MA, Ogura Y, Krause G, Phillips AD, Gomes TA, Hayashi T, Frankel G 2007 TccP2-mediated subversion of actin dynamics by EPEC 2: a distinct evolutionary lineage of enteropathogenic *Escherichia coli*. Microbiology 153:1743–1755. doi:10.1099/mic.0.2006/004325-0. 17526832 PMC2884950

[45] Battle SE, Brady MJ, Vanaja SK, Leong JM, Hecht GA 2014 Actin pedestal formation by enterohemorrhagic *Escherichia coli* enhances bacterial host cell attachment and concomitant type III translocation. Infect Immun 82:3713–3722. doi:10.1128/IAI.01523-13. 24958711 PMC4187837

[46] Bai L, Schuller S, Whale A, Mousnier A, Marches O, Wang L, Ooka T, Heuschkel R, Torrente F, Kaper JB, Gomes TA, Xu J, Phillips AD, Frankel G 2008 Enteropathogenic *Escherichia coli* O125:H6 triggers attaching and effacing lesions on human intestinal biopsy specimens independently of Nck and TccP/TccP2. Infect Immun 76:361–368. doi:10.1128/IAI.01199-07. 17984209 PMC2223649

[47] Ritchie JM, Brady MJ, Riley KN, Ho TD, Campellone KG, Herman IM, Donohue-Rolfe A, Tzipori S, Waldor MK, Leong JM 2008 EspFU, a type III-translocated effector of actin assembly, fosters epithelial association and late-stage intestinal colonization by *Escherichia coli* O157:H7. Cell Microbiol 10:836–847. doi:10.1111/j.1462-5822.2007.01087.x. 18067584 PMC2504705

[48] Velle KB, Campellone KG 2017 Extracellular motility and cell-to-cell transmission of enterohemorrhagic *E. coli* is driven by EspFU-mediated actin assembly. PLoS Pathog 13:e1006501. doi:10.1371/journal.ppat.1006501. 28771584 PMC5557606

[49] Gruber CC, Sperandio V 2014 Posttranscriptional control of microbe-induced rearrangement of host cell actin. mBio 5:e01025-13. doi:10.1128/mBio.01025-13. 24425733 PMC3903284

[50] Lai Y, Rosenshine I, Leong JM, Frankel G 2013 Intimate host attachment: enteropathogenic and enterohaemorrhagic *Escherichia coli*. Cell Microbiol 15:1796–1808. doi:10.1111/cmi.12179. 23927593 PMC4036124

[51] Gruenheid S, DeVinney R, Bladt F, Goosney D, Gelkop S, Gish GD, Pawson T, Finlay BB 2001 Enteropathogenic *E. coli* Tir binds Nck to initiate actin pedestal formation in host cells. Nat Cell Biol 3:856–859. doi:10.1038/ncb0901-856. 11533668

[52] Rohatgi R, Nollau P, Ho HY, Kirschner MW, Mayer BJ 2001 Nck and phosphatidylinositol 4,5-bisphosphate synergistically activate actin polymerization through the N-WASP-Arp2/3 pathway. J Biol Chem 276:26448–26452. doi:10.1074/jbc.M103856200. 11340081

[53] Nobe R, Nougayrede JP, Taieb F, Bardiau M, Cassart D, Navarro-Garcia F, Mainil J, Hayashi T, Oswald E 2009 Enterohaemorrhagic *Escherichia coli* serogroup O111 inhibits NF-κB-dependent innate responses in a manner independent of a type III secreted OspG orthologue. Microbiology 155:3214–3225. doi:10.1099/mic.0.030759-0. 19628559

[54] Ruchaud-Sparagano MH, Maresca M, Kenny B 2007 Enteropathogenic *Escherichia coli* (EPEC) inactivate innate immune responses prior to compromising epithelial barrier function. Cell Microbiol 9:1909–1921. doi:10.1111/j.1462-5822.2007.00923.x. 17388785 PMC1974805

[55] Savkovic SD, Koutsouris A, Hecht G 1997 Activation of NF-κB in intestinal epithelial cells by enteropathogenic *Escherichia coli*. Am J Physiol 273:C1160–7. doi:10.1152/ajpcell.1997.273.4.C1160. 9357759

[56] Sharma R, Tesfay S, Tomson FL, Kanteti RP, Viswanathan VK, Hecht G 2006 Balance of bacterial pro- and anti-inflammatory mediators dictates net effect of enteropathogenic *Escherichia coli* on intestinal epithelial cells. Am J Physiol Gastrointest Liver Physiol 290:G685–G694. doi:10.1152/ajpgi.00404.2005. 16322091

[57] Hauf N, Chakraborty T 2003 Suppression of NF-κB activation and proinflammatory cytokine expression by Shiga toxin-producing *Escherichia coli*. J Immunol 170:2074–2082. doi:10.4049/jimmunol.170.4.2074. 12574378

[58] Elbaz N, Socol Y, Katsowich N, Rosenshine I 2019 Control of type III secretion system effector/chaperone ratio fosters pathogen adaptation to host-adherent lifestyle. mBio 10:e02074-19. doi:10.1128/mBio.02074-19. 31530678 PMC6751064

[59] Mallick EM, Garber JJ, Vanguri VK, Balasubramanian S, Blood T, Clark S, Vingadassalom D, Louissaint C, McCormick B, Snapper SB, Leong JM 2014 The ability of an attaching and effacing pathogen to trigger localized actin assembly contributes to virulence by promoting mucosal attachment. Cell Microbiol 16:1405–1424. doi:10.1111/cmi.12302. 24780054 PMC4146666

[60] Datsenko KA, Wanner BL 2000 One-step inactivation of chromosomal genes in *Escherichia coli* K-12 using PCR products. Proc Natl Acad Sci U S A 97:6640–6645. doi:10.1073/pnas.120163297. 10829079 PMC18686

[61] Bolger AM, Lohse M, Usadel B 2014 Trimmomatic: a flexible trimmer for Illumina sequence data. Bioinformatics 30:2114–2120. doi:10.1093/bioinformatics/btu170. 24695404 PMC4103590

[62] Lin SH, Liao YC 2013 CISA: contig integrator for sequence assembly of bacterial genomes. PLoS One 8:e60843. doi:10.1371/journal.pone.0060843. 23556006 PMC3610655

[63] Jackman SD, Vandervalk BP, Mohamadi H, Chu J, Yeo S, Hammond SA, Jahesh G, Khan H, Coombe L, Warren RL, Birol I 2017 ABySS 2.0: resource-efficient assembly of large genomes using a Bloom filter. Genome Res 27:768–777. doi:10.1101/gr.214346.116. 28232478 PMC5411771

[64] Zerbino DR 2010 Using the Velvet *de novo* assembler for short-read sequencing technologies. Curr Protoc Bioinform 31:11.5.1–11.5.12. 10.1002/0471250953.bi1105s31 PMC2952100 20836074

[65] Luo R, Liu B, Xie Y, Li Z, Huang W, Yuan J, He G, Chen Y, Pan Q, Liu Y, Tang J, Wu G, Zhang H, Shi Y, Liu Y, Yu C, Wang B, Lu Y, Han C, Cheung DW, Yiu SM, Peng S, Xiaoqian Z, Liu G, Liao X, Li Y, Yang H, Wang J, Lam TW, Wang J 2012 SOAPdenovo2: an empirically improved memory-efficient short-read *de novo* assembler. Gigascience 1:18. doi:10.1186/2047-217X-1-18. 23587118 PMC3626529

[66] Bankevich A, Nurk S, Antipov D, Gurevich AA, Dvorkin M, Kulikov AS, Lesin VM, Nikolenko SI, Pham S, Prjibelski AD, Pyshkin AV, Sirotkin AV, Vyahhi N, Tesler G, Alekseyev MA, Pevzner PA 2012 SPAdes: a new genome assembly algorithm and its applications to single-cell sequencing. J Comput Biol 19:455–477. doi:10.1089/cmb.2012.0021. 22506599 PMC3342519

[67] Rozov R, Brown Kav A, Bogumil D, Shterzer N, Halperin E, Mizrahi I, Shamir R 2017 Recycler: an algorithm for detecting plasmids from *de novo* assembly graphs. Bioinformatics 33:475–482. doi:10.1093/bioinformatics/btw651. 28003256 PMC5408804

[68] Roosaare M, Puustusmaa M, Mols M, Vaher M, Remm M 2018 PlasmidSeeker: identification of known plasmids from bacterial whole-genome sequencing reads. PeerJ 6:e4588. doi:10.7717/peerj.4588. 29629246 PMC5885972

[69] Seemann T 2014 Prokka: rapid prokaryotic genome annotation. Bioinformatics 30:2068–2069. doi:10.1093/bioinformatics/btu153. 24642063

[70] Rasko DA, Myers GS, Ravel J 2005 Visualization of comparative genomic analyses by BLAST score ratio. BMC Bioinformatics 6:2. doi:10.1186/1471-2105-6-2. 15634352 PMC545078

[71] Hazen TH, Sahl JW, Fraser CM, Donnenberg MS, Scheutz F, Rasko DA 2013 Refining the pathovar paradigm via phylogenomics of the attaching and effacing *Escherichia coli*. Proc Natl Acad Sci U S A 110:12810–12815. doi:10.1073/pnas.1306836110. 23858472 PMC3732946

[72] Tobe T, Beatson SA, Taniguchi H, Abe H, Bailey CM, Fivian A, Younis R, Matthews S, Marches O, Frankel G, Hayashi T, Pallen MJ 2006 An extensive repertoire of type III secretion effectors in *Escherichia coli* O157 and the role of lambdoid phages in their dissemination. Proc Natl Acad Sci U S A 103:14941–14946. doi:10.1073/pnas.0604891103. 16990433 PMC1595455

[73] Iguchi A, Thomson NR, Ogura Y, Saunders D, Ooka T, Henderson IR, Harris D, Asadulghani M, Kurokawa K, Dean P, Kenny B, Quail MA, Thurston S, Dougan G, Hayashi T, Parkhill J, Frankel G 2009 Complete genome sequence and comparative genome analysis of enteropathogenic *Escherichia coli* O127:H6 strain E2348/69. J Bacteriol 191:347–354. doi:10.1128/JB.01238-08. 18952797 PMC2612414

[74] Deng W, Yu HB, de Hoog CL, Stoynov N, Li Y, Foster LJ, Finlay BB 2012 Quantitative proteomic analysis of type III secretome of enteropathogenic *Escherichia coli* reveals an expanded effector repertoire for attaching/effacing bacterial pathogens. Mol Cell Proteomics 11:692–709. doi:10.1074/mcp.M111.013672. 22661456 PMC3434768

[75] Ogura Y, Abe H, Katsura K, Kurokawa K, Asadulghani M, Iguchi A, Ooka T, Nakayama K, Yamashita A, Hattori M, Tobe T, Hayashi T 2008 Systematic identification and sequence analysis of the genomic islands of the enteropathogenic *Escherichia coli* strain B171-8 by the combined use of whole-genome PCR scanning and fosmid mapping. J Bacteriol 190:6948–6960. doi:10.1128/JB.00625-08. 18757547 PMC2580699

[76] Arndt D, Grant JR, Marcu A, Sajed T, Pon A, Liang Y, Wishart DS 2016 PHASTER: a better, faster version of the PHAST phage search tool. Nucleic Acids Res 44:W16–W21. doi:10.1093/nar/gkw387. 27141966 PMC4987931

[77] Sullivan MJ, Petty NK, Beatson SA 2011 Easyfig: a genome comparison visualizer. Bioinformatics 27:1009–1010. doi:10.1093/bioinformatics/btr039. 21278367 PMC3065679

[78] Sievers F, Wilm A, Dineen D, Gibson TJ, Karplus K, Li W, Lopez R, McWilliam H, Remmert M, Soding J, Thompson JD, Higgins DG 2011 Fast, scalable generation of high-quality protein multiple sequence alignments using Clustal Omega. Mol Syst Biol 7:539. doi:10.1038/msb.2011.75. 21988835 PMC3261699

[79] Knutton S, Baldwin T, Williams PH, McNeish AS 1989 Actin accumulation at sites of bacterial adhesion to tissue culture cells: basis of a new diagnostic test for enteropathogenic and enterohemorrhagic *Escherichia coli*. Infect Immun 57:1290–1298. doi:10.1128/IAI.57.4.1290-1298.1989. 2647635 PMC313264

[80] Knutton S 1995 Electron microscopical methods in adhesion. Methods Enzymol 253:145–158. doi:10.1016/s0076-6879(95)53015-0. 7476383

[81] Kim D, Pertea G, Trapnell C, Pimentel H, Kelley R, Salzberg SL 2013 TopHat2: accurate alignment of transcriptomes in the presence of insertions, deletions and gene fusions. Genome Biol 14:R36. doi:10.1186/gb-2013-14-4-r36. 23618408 PMC4053844

[82] Liao Y, Smyth GK, Shi W 2014 featureCounts: an efficient general purpose program for assigning sequence reads to genomic features. Bioinformatics 30:923–930. doi:10.1093/bioinformatics/btt656. 24227677

[83] Robinson MD, McCarthy DJ, Smyth GK 2010 edgeR: a Bioconductor package for differential expression analysis of digital gene expression data. Bioinformatics 26:139–140. doi:10.1093/bioinformatics/btp616. 19910308 PMC2796818

[84] Gu Z, Eils R, Schlesner M 2016 Complex heatmaps reveal patterns and correlations in multidimensional genomic data. Bioinformatics 32:2847–2849. doi:10.1093/bioinformatics/btw313. 27207943

