# Supplementary figures and images for: EspFu-Mediated Actin Assembly Enhances Enteropathogenic Escherichia coli Adherence and Activates Host Cell Inflammatory Signaling Pathways
Source: mBio. 2020 Apr 14;11(2):e00617-20. doi: 10.1128/mBio.00617-20 (PMC7157822; doi:10.1128/mBio.00617-20)

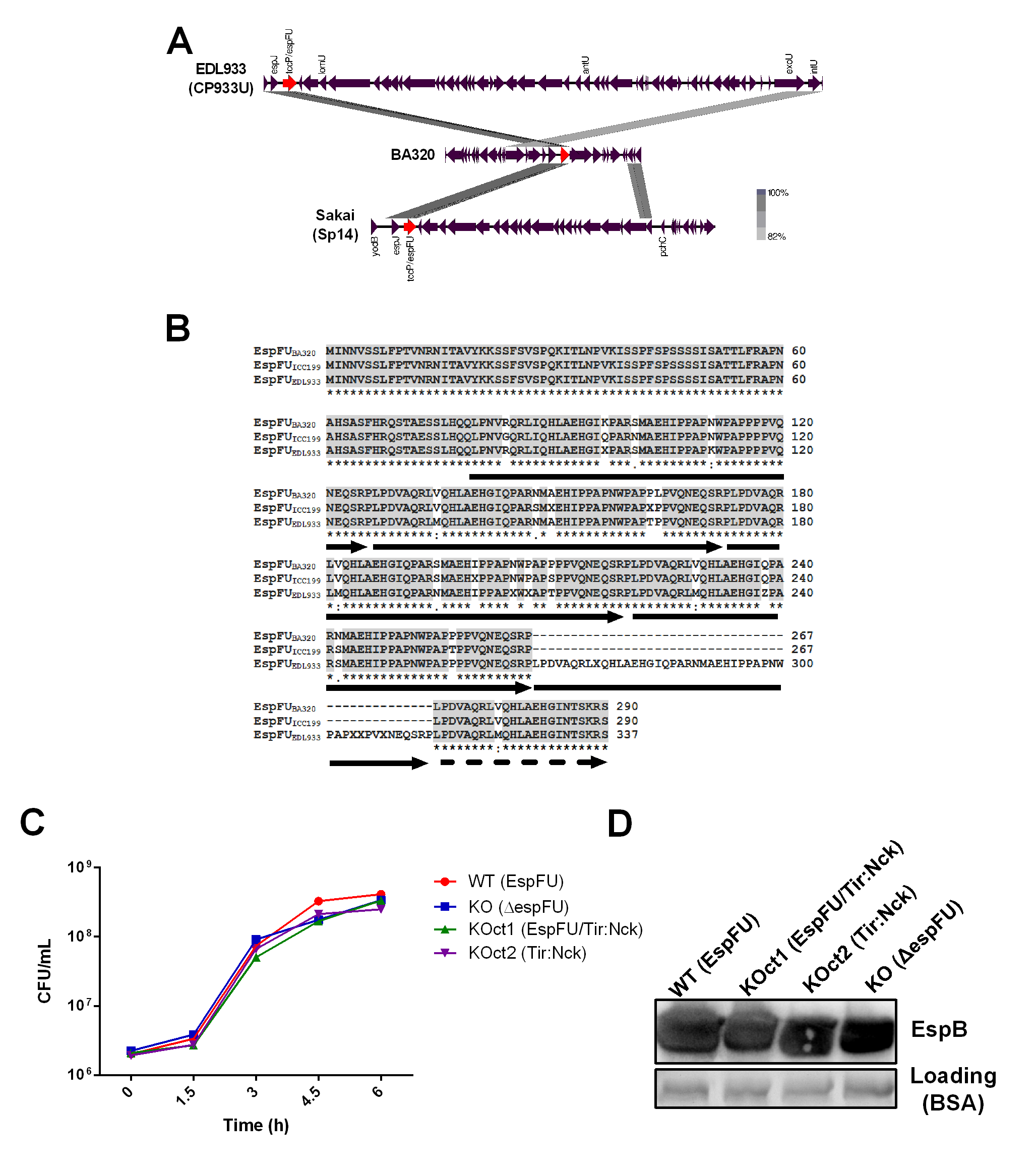

Supplement: FIG S1 [file mBio.00617-20-sf001.tif]

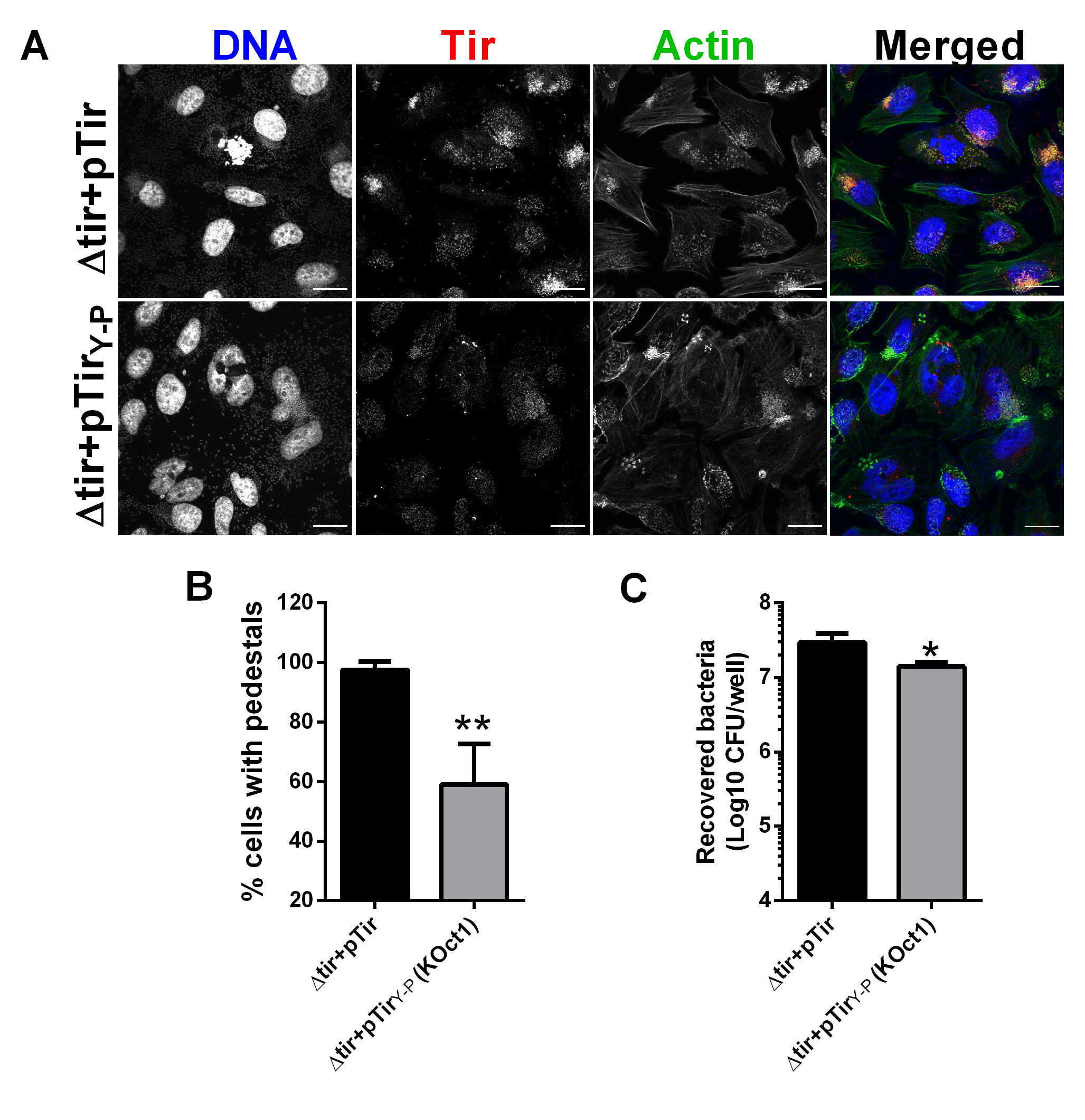

Supplement: FIG S2 [file mBio.00617-20-sf002.tif]

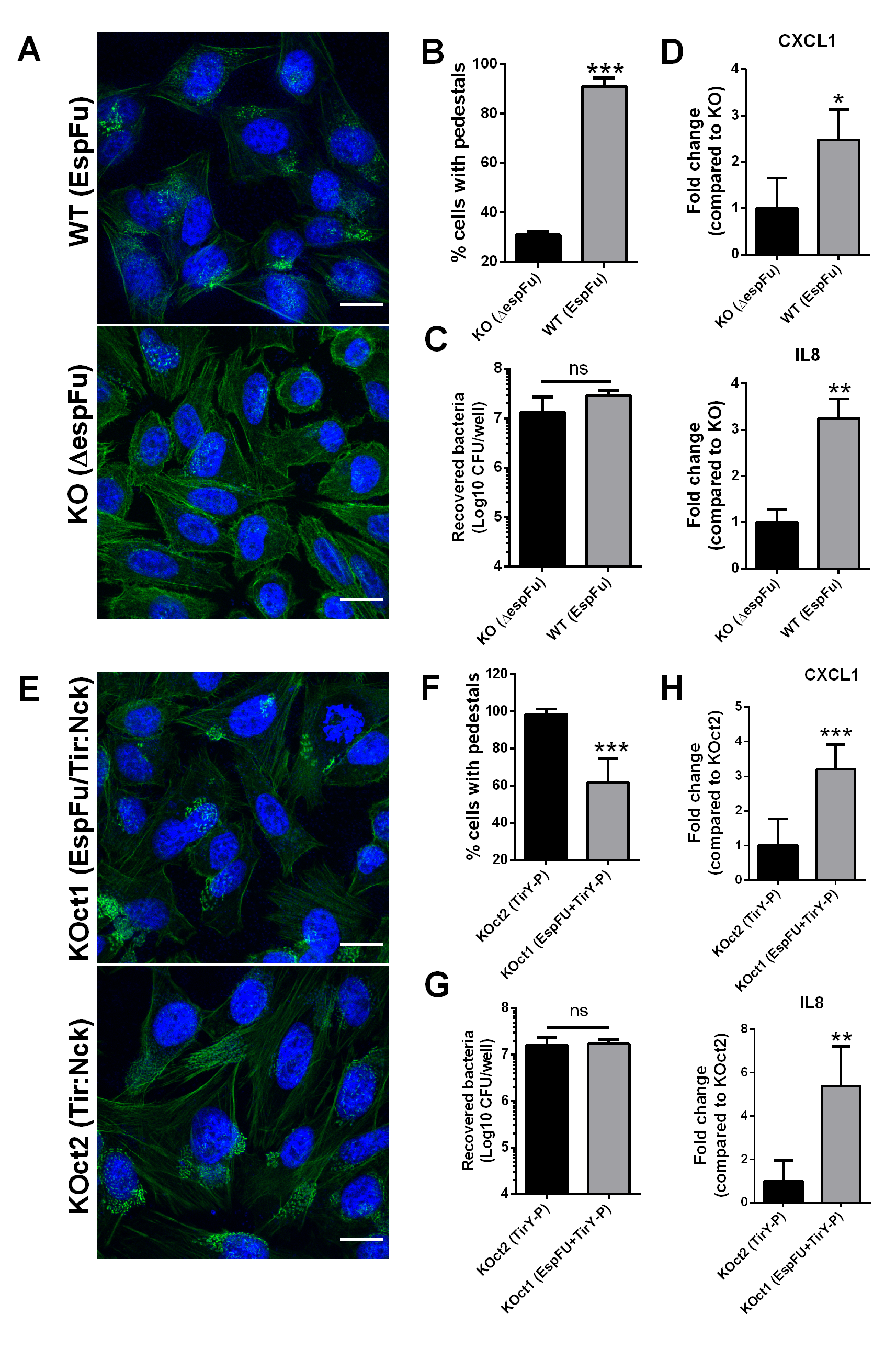

Supplement: FIG S3 [file mBio.00617-20-sf003.tif]

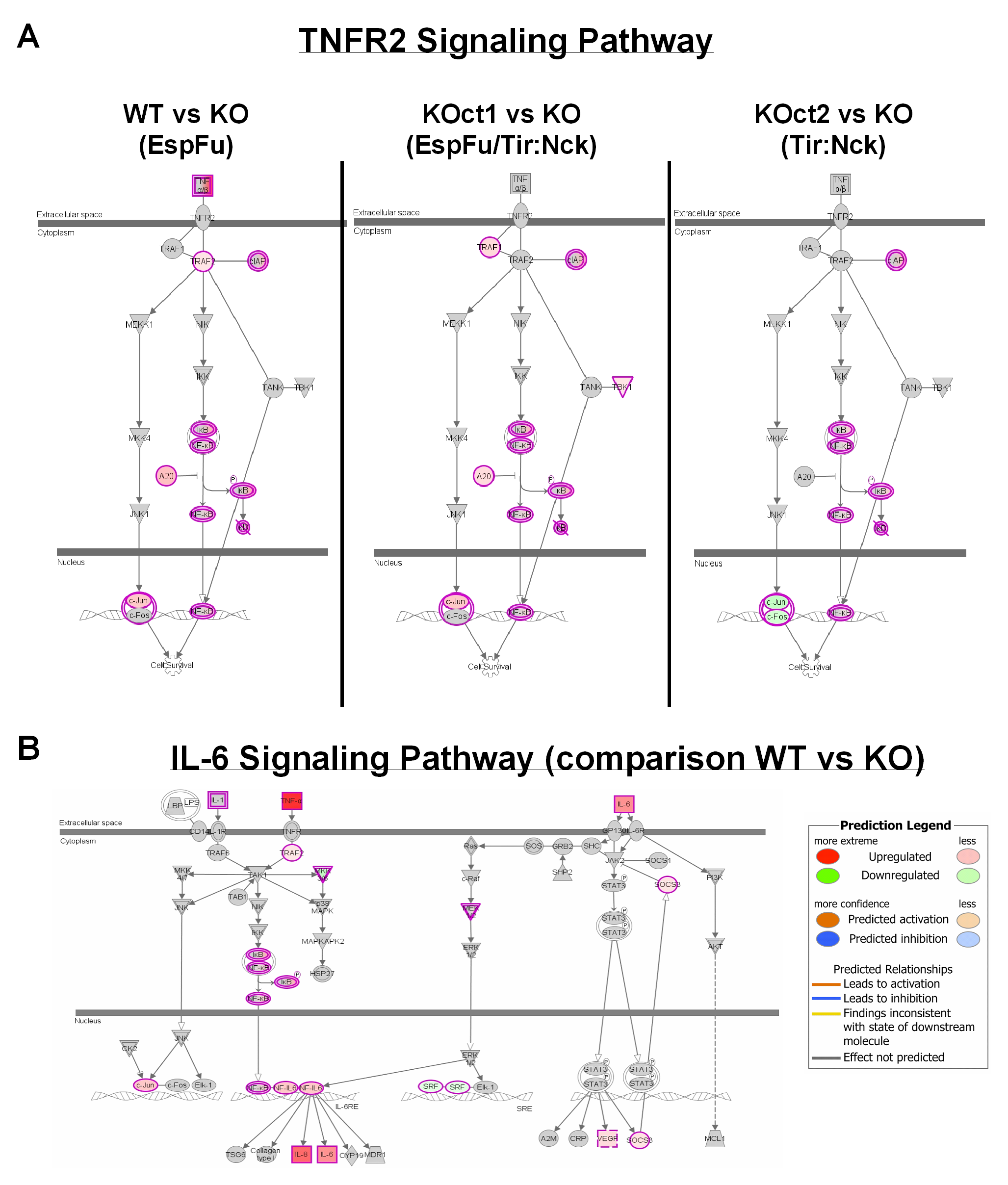

Supplement: FIG S4 [file mBio.00617-20-sf004.tif]

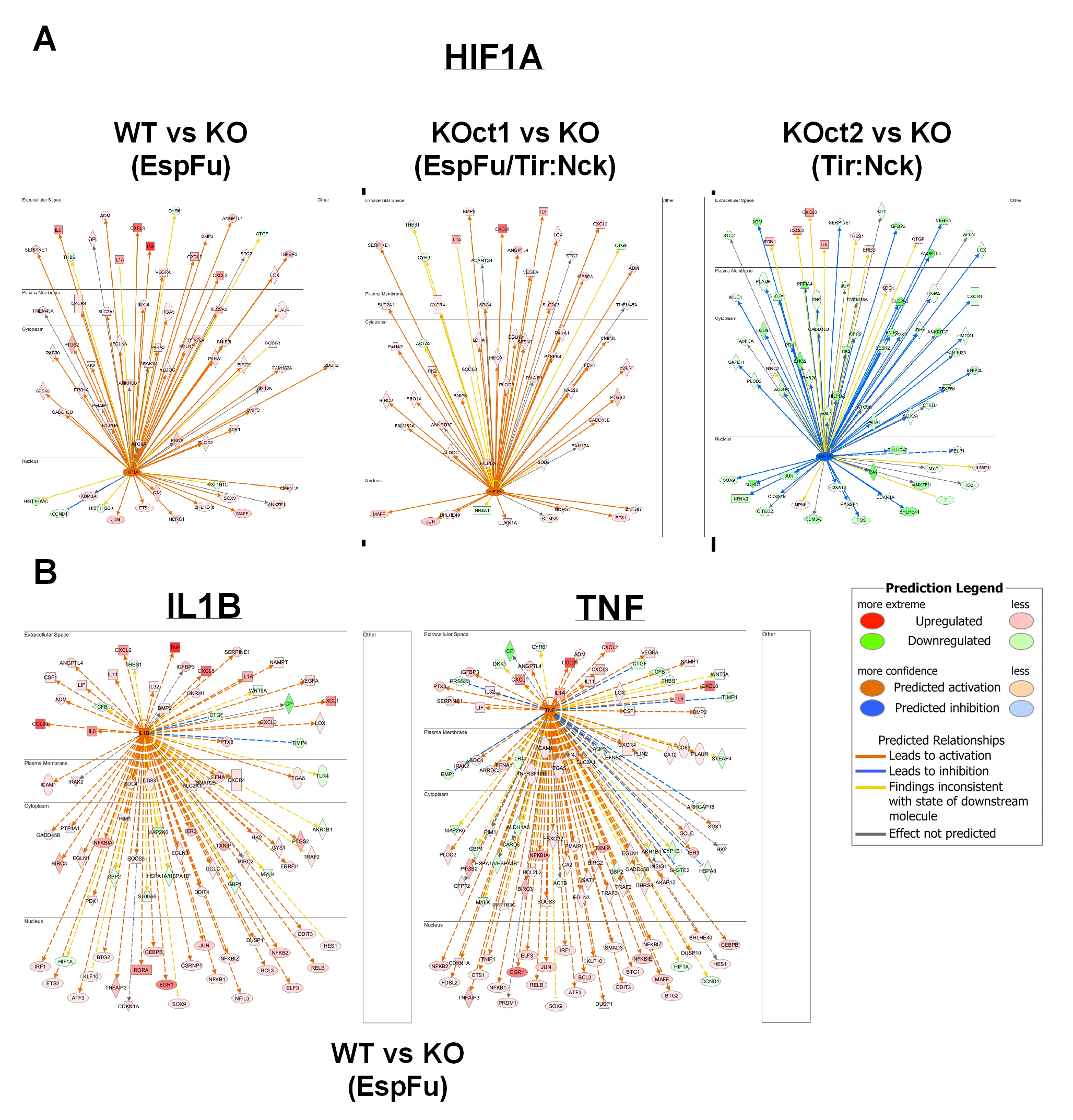

Supplement: FIG S5 [file mBio.00617-20-sf005.tif]
