# Supplementary material for: EspFu-Mediated Actin Assembly Enhances Enteropathogenic Escherichia coli Adherence and Activates Host Cell Inflammatory Signaling Pathways
Source: mBio. 2020 Apr 14;11(2):e00617-20. doi: 10.1128/mBio.00617-20 (PMC7157822; doi:10.1128/mBio.00617-20)
Supplement: TABLE S1 [file mBio.00617-20-st001.docx]

**Table S1 – Bacterial strains, plasmids and oligonucleotide primers used in this study.**

| **Strains** | | **Relevant description** | **Reference or source** | |
| --- | --- | --- | --- | --- |
| BA320 (WT) | | wild-type atypical EPEC O55:H7 | (1, 2) | |
| ΔespFu (KO) | | BA320 *espFu* nonpolar mutant | This study | |
| KO+pEspFu | | ΔespFu (KO) strain complemented with pEspFu | This study | |
| KO+vector | | ΔespFu (KO) strain with pACYC184 (empty vector) | This study | |
| Δtir | | BA320 *tir* nonpolar mutant | This study | |
| Δtir+pTir | | Δtir strain complemented with pTir | This study | |
| Δtir+pTir_Y-P_ (KOct1) | | Δtir strain complemented with pEP23 (pTir_Y-P_) | This study | |
| ΔΔespFu:tir | | BA320 *espFu-tir* double nonpolar mutant | This study | |
| ΔΔespFu:tir+pTir_Y-P_ (KOct2) | | ΔΔespFu:tir strain complemented with pEP23 (pTir_Y-P_) | This study | |
| E2348/69 | | Prototype EPEC strain (serotype O127:H6) | (3) | |
| DH5α | | *E. coli* K-12 strain for plasmid propagation | Thermo Fisher | |
|  | |  |  | |
| **Plasmids** | |  |  | |
| pKD46 | | λ red helper plasmid | (4) | |
| pKD3 | | λ red template plasmid | (4) | |
| pKD4 | | λ red template plasmid | (4) | |
| pCP20 | | λ red resolvase plasmid | (4) | |
| pACYC184 | | Low-copy-number cloning vector | New England Biolabs | |
| pEspFu | | BA320 *espFU* under P_tet_ promoter in pACYC184 | This study | |
| pEP23 (pTir_Y-P_) | | E2348/69 *tir* under P_tet_ promoter in pACYC184 | (5) | |
| pDP151 | | mCherry-expressing plasmid | (6) | |
|  |  | | |  |
| **Primer** | **Sequence (5'-3')** | | |  |
| **Nonpolar mutant construction (Lambda Red)** | | | |  |
| EspFUcat-1 | ATGATTAACAATGTTTCTTCACTTTTTCCAACCGTCAACCGCAATATTACAGCTGTATAT | | |  |
|  | GTGTAGGCTGGAGCTGCTTC | | |  |
| EspFUcat-2 | TCACGAGCGCTTAGATGTATTAATGCCATGCTCTGCAAGATGCTGCACCAGACGCTGAGC | | |  |
|  | CATATGAATATCCTCCTTAG | | |  |
| Tirkan-1 | ATGCCTATTGGTAATCTTGGTCATAATCCCAATGTGAATAATTCAATTCCTCCTGCACCT | | |  |
|  | GTGTAGGCTGGAGCTGCTTC | | |  |
| Tirkan-2 | GGATCCCGGCGCTGGTGGGTTATTCGAAGTATTCACAGCGCTATTACTCCCCCCCGTTAA | | |  |
|  | CATATGAATATCCTCCTTAG | | |  |
| **Complementation of EspFU and Tir (cloning in pACYC184)** | | | |  |
| EspFU-F2 | CATGGATCCATGATTAACAATGTTTCTTCAC | | |  |
| EspFU-R2 | CTAGTGGACTCACGAGCGCTTAGATGTATTAATG | | |  |
| Tir004+ | GGAAGATCTAGGAAAGGAGARATTTATGCCTATTGG | | |  |
| Tir005- | CGGCCTGGATATATTTAGACGAAACG | | |  |
| **qRT-PCR (RNA-seq validation)** | | | |  |
| B2M-F | CTATCCAGCGTACTCCAAAG | | |  |
| B2M-R | GAAAGACCAGTCCTTGCTGA | | |  |
| CXCL1-F | AACCGAAGTCATAGCCACAC | | |  |
| CXCL1-R | CCTCCCTTCTGGTCAGTTG | | |  |
| IL6-F | CCACTCACCTCTTCAGAACG | | |  |
| IL6-R | CATCTTTGGAAGGTTCAGGTTG | | |  |
| IL8-F | ATACTCCAAACCTTTCCACCC | | |  |
| IL8-R | TCTGCACCCAGTTTTCCTTG | | |  |
| NFKBIA-F | ACCTGGTGTCACTCCTGTTGA | | |  |
| NFKBIA-R | CTGCTGCTGTATCCGGGTG | | |  |

**REFERENCES**

1. Abe CM, Trabulsi LR, Blanco J, Blanco M, Dahbi G, Blanco JE, Mora A, Franzolin MR, Taddei CR, Martinez MB, Piazza RM, Elias WP. 2009. Virulence features of atypical enteropathogenic Escherichia coli identified by the eae(+) EAF-negative stx(-) genetic profile. Diagn Microbiol Infect Dis 64:357-65.

2. Bueris V, Sircili MP, Taddei CR, dos Santos MF, Franzolin MR, Martinez MB, Ferrer SR, Barreto ML, Trabulsi LR. 2007. Detection of diarrheagenic Escherichia coli from children with and without diarrhea in Salvador, Bahia, Brazil. Mem Inst Oswaldo Cruz 102:839-44.

3. Levine MM, Bergquist EJ, Nalin DR, Waterman DH, Hornick RB, Young CR, Sotman S. 1978. Escherichia coli strains that cause diarrhoea but do not produce heat-labile or heat-stable enterotoxins and are non-invasive. Lancet 1:1119-22.

4. Datsenko KA, Wanner BL. 2000. One-step inactivation of chromosomal genes in Escherichia coli K-12 using PCR products. Proc Natl Acad Sci U S A 97:6640-5.

5. DeVinney R, Puente JL, Gauthier A, Goosney D, Finlay BB. 2001. Enterohaemorrhagic and enteropathogenic Escherichia coli use a different Tir-based mechanism for pedestal formation. Mol Microbiol 41:1445-58.

6. Gruber CC, Sperandio V. 2014. Posttranscriptional control of microbe-induced rearrangement of host cell actin. MBio 5:e01025-13.
