# Supplementary material for: EspFu-Mediated Actin Assembly Enhances Enteropathogenic Escherichia coli Adherence and Activates Host Cell Inflammatory Signaling Pathways
Source: mBio. 2020 Apr 14;11(2):e00617-20. doi: 10.1128/mBio.00617-20 (PMC7157822; doi:10.1128/mBio.00617-20)
Supplement: TABLE S2 [file mBio.00617-20-st002.docx]

| **Table S2 – List of differentially expressed genes (DEGs) in each comparison.** | | | | | | |
| --- | --- | --- | --- | --- | --- | --- |
|  |  |  | |  |  |  |
| **Comparison (log2FC)** | | | | **Gene symbol** | **Gene name** | **Relevant activity/description** |
| **WT x KO** | **KOct1 x KO** | | **KOct2 x KO** |  |  |  |
| 0.48 | 0.45 | | -1.87 | ADM | adrenomedullin | signaling molecule (hormone) |
| -0.28 | 2.50 | | 0.23 | AKR1B10 | aldo-keto reductase family 1 member B10 | oxidoreductase |
| -3.69* | 9.12 | | 1.63* | ALDH1A1 | aldehyde dehydrogenase 1 family member A1 | oxidoreductase |
| 0.48 | -0.69 | | -1.33 | AMH | anti-Mullerian hormone | signaling molecule (growth factor) |
| 0.93 | 1.01 | | -2.48 | ANGPTL4 | angiopoietin like 4 | angiogenesis/developmental protein/differentiation/extracellular matrix/signaling molecule |
| -1.45 | -0.76 | | 0.52 | ANKRD1 | ankyrin repeat domain 1 | nucleic acid binding |
| 0.78 | 0.74 | | -1.87 | ANKRD37 | ankyrin repeat domain 37 | nucleic acid binding |
| 0.80 | 0.10 | | -1.32 | ANKZF1 | ankyrin repeat and zinc finger domain containing 1 | nucleic acid binding |
| 0.50 | 0.43 | | -1.48 | BHLHE40 | basic helix-loop-helix family member e40 | nucleic acid binding/transcription regulation |
| 0.50 | 0.25 | | -1.88 | BHLHE41 | basic helix-loop-helix family member e41 | nucleic acid binding/transcription regulation |
| 1.43 | 1.47 | | 1.11 | BIRC3 | baculoviral IAP repeat containing 3 | transferase/apoptosis |
| 0.74 | 0.52 | | -1.47 | BNIP3 | BCL2 interacting protein 3 | host-virus interaction/membrane protein/apoptosis |
| 0.72 | 0.63 | | -1.12 | BNIP3L | BCL2 interacting protein 3 like | host-virus interaction/membrane protein/apoptosis |
| 0.09 | -0.15 | | -1.04 | C19orf26 (CBARP) | CACN beta subunit associated regulatory protein | exocytosis/synapse |
| 0.46 | 0.18 | | -1.31 | C1orf51 (CIART) | circadian associated repressor of transcription | transcription regulation |
| 1.38 | 1.03 | | 0.38 | C8orf4 (TCIM) | chromosome 8 open reading frame 4 | apoptosis |
| 0.95 | 0.44 | | -2.41 | CA9 | carbonic anhydrase 9 | lyase |
| 0.20 | 0.21 | | 1.30 | CCL2 | C-C motif chemokine ligand 2 | signaling molecule (cytokine)/chemotaxis/inflammatory response |
| 0.96 | 0.76 | | -1.19 | CCNG2 | cyclin G2 | cell cycle (cyclin) |
| 1.06 | 0.68 | | -0.52 | CEBPB | CCAAT/enhancer binding protein beta | nucleic acid binding/transcription regulation/inflammatory response |
| 0.39 | 0.45 | | -1.06 | CHAC1 | ChaC glutathione specific gamma-glutamylcyclotransferase 1 | transferase (acyltransferase) |
| -0.15 | -0.17 | | -1.21 | COL9A3 | collagen type IX alpha 3 chain | extracellular matrix (collagen) |
| 2.00 | 1.75 | | 1.88 | CXCL1 | C-X-C motif chemokine ligand 1 | signaling molecule (cytokine)/inflammatory response |
| 1.80 | 0.92 | | 1.28 | CXCL2 | C-X-C motif chemokine ligand 2 | signaling molecule (cytokine)/chemotaxis/inflammatory response |
| 1.15 | 0.89 | | 0.57 | CXCL3 | C-X-C motif chemokine ligand 3 | signaling molecule (cytokine)/chemotaxis/inflammatory response |
| -0.29 | -0.03 | | -1.10 | DBP | D-box binding PAR bZIP transcription factor | nucleic acid binding/transcription regulation |
| 0.36 | 0.37 | | -1.62 | DDIT4 | DNA damage inducible transcript 4 | apoptosis/antiviral defense |
| -0.22 | -0.39 | | -3.35 | EDN2 | endothelin 2 | signaling molecule (vasoactive) |
| 1.07 | 0.65 | | -0.96 | EFNA1 | ephrin A1 | signaling molecule/tumor suppressor/angiogenesis |
| 0.72 | 0.32 | | -1.93 | EFNA3 | ephrin A3 | signaling molecule |
| 0.55 | 0.45 | | -1.62 | EGLN3 | egl-9 family hypoxia inducible factor 3 | oxidoreductase/apoptosis |
| 2.34 | 1.94 | | 0.78 | EGR1 | early growth response 1 | nucleic acid binding/transcription regulation |
| 1.16 | 0.40 | | -2.10 | ENO2 | enolase 2 | lyase |
| 1.10 | 1.14 | | -1.38 | FAM110C | family with sequence similarity 110 member C | cytoskeletal protein |
| 0.87 | 0.72 | | -2.14 | FAM115C (TCAF2) | TRPM8 channel-associated factor 2 | transport |
| -0.06 | 0.24 | | -1.13 | FAM212A | family with sequence similarity 210 member A | developmental protein |
| -1.73 | -0.10 | | -0.43 | FBXO2 | F-box protein 2 | ubiquitination |
| 0.62 | 0.34 | | -1.81 | FGF11 | fibroblast growth factor 11 | signaling molecule (growth factor) |
| 1.04 | 0.57 | | 0.97 | FGFR2 | fibroblast growth factor receptor 2 | apoptosis/cell proliferation/differentiation/receptor/transferase (kinase) |
| 0.91 | 0.43 | | -2.36 | FOXD1 | forkhead box D1 | nucleic acid binding/transcription regulation |
| 0.66 | 0.41 | | -1.43 | FUT11 | fucosyltransferase 11 | transferase (glycosyltransferase) |
| 0.52 | 0.30 | | -1.25 | FZD8 | frizzled class receptor 8 | developmental protein/signal transduction/receptor |
| 0.98 | 1.21 | | -1.40 | GDF15 | growth differentiation factor 15 | signaling molecule (cytokine/growth factor) |
| 0.10 | -0.92 | | -1.02 | GNB3 | G protein subunit beta 3 | signal transduction |
| 0.13 | -0.57 | | -1.03 | GPC2 | glypican 2 | extracellular matrix (proteoglycan) |
| 1.34 | 1.36 | | -2.69 | GPR146 | G protein-coupled receptor 146 | signal transduction/receptor |
| 0.48 | 0.40 | | -1.01 | HES1 | hes family bHLH transcription factor 1 | nucleic acid binding/transcription regulation |
| 0.99 | 0.19 | | -1.00 | HES7 | hes family bHLH transcription factor 7 | developmental protein/nucleic acid binding/transcription regulation |
| 0.74 | 0.55 | | -1.25 | HEY1 | hes related family bHLH transcription factor with YRPW motif 1 | developmental protein/nucleic acid binding/transcription regulation |
| 0.99 | 0.79 | | -1.69 | HILPDA | hypoxia inducible lipid droplet associated | signaling molecule/stress response |
| 0.69 | 0.49 | | -1.51 | HK2 | hexokinase 2 | transferase (kinase) |
| 0.57 | -0.06 | | -1.30 | ICAM5 | intercellular adhesion molecule 5 | cell adhesion |
| 1.21 | 1.17 | | -0.88 | IER3 | immediate early response 3 | apoptosis |
| 1.15 | 0.79 | | -1.36 | IGFBP3 | insulin-like growth factor binding protein 3 | signaling molecule/apoptosis |
| 1.53 | 1.66 | | 0.31 | IL1A | interleukin 1 alpha | signaling molecule (cytokine)/inflammatory response/pyrogen |
| 2.00 | 1.25 | | 1.28 | IL6 | interleukin 6 | signaling molecule (cytokine/growth factor)/inflammatory response |
| 2.69 | 2.74 | | 1.51 | IL8 (CXCL8) | interleukin 8 (C-X-C motif chemokine ligand 8) | signaling molecule (cytokine)/chemotaxis/inflammatory response |
| 1.00 | 0.70 | | -1.05 | INSIG2 | insulin induced gene 2 | lipid metabolism |
| 0.98 | 1.06 | | 0.78 | IRAK2 | interleukin 1 receptor associated kinase 2 | signal transduction/inflammatory response |
| -0.50 | -0.57 | | -1.10 | ISYNA1 | inositol-3-phosphate synthase 1 | isomerase (inositol phosphate metabolism) |
| -0.03 | -0.76 | | -1.15 | IZUMO4 | IZUMO family member 4 | signaling molecule (glycoprotein) |
| 1.24 | 1.03 | | -0.52 | JHDM1D (KDM7A) | lysine-specific demethylase 7A | oxidoreductase/transcription regulation |
| 1.07 | 1.06 | | -1.05 | JUN | Jun proto-oncogene, AP-1 transcription factor subunit | nucleic acid binding/transcription regulation/proto-oncogene |
| 1.01* | 1.04* | | -1.29 | JUNB | JunB proto-oncogene, AP-1 transcription factor subunit | hydrolase/nucleic acid binding/transcription regulation |
| 1.07 | 0.91 | | -1.58 | KCTD11 | potassium channel tetramerization domain containing 11 | cell cycle/developmental protein/ion transport/tumor suppressor |
| 0.68 | 0.48 | | -1.19 | KDM3A | lysine demethylase 3A | oxidoreductase/transcription regulation |
| 0.80 | 0.35 | | -1.12 | KDM6B | lysine demethylase 6B | oxidoreductase |
| -1.01 | -0.33 | | -0.09 | KRT34 | keratin 34 | structural activity (keratinization) |
| 0.20 | -0.71 | | -1.16 | LENG8 | leukocyte receptor cluster member 8 | receptor |
| -0.09 | -0.83 | | -1.14 | LINC00115 | long intergenic non-protein coding RNA 115 | non-protein coding RNA |
| 0.04 | -0.49 | | -1.24 | LINC00482 | long intergenic non-protein coding RNA 482 | non-protein coding RNA |
| 1.02 | 1.13 | | -0.37 | LINC00673 | long intergenic non-protein coding RNA 673 | non-protein coding RNA/tumor progression |
| -0.12 | -1.33 | | -0.60 | LOC100130557 (NFYC-AS1) | NFYC antisense RNA 1 | non-proteing coding RNA |
| -0.63 | -1.12 | | -0.84 | LOC100132356 | uncharacterized LOC100132356 | non-proteing coding RNA |
| 0.55 | 0.34 | | -1.03 | LOC100134229 (JHDM1D-AS1) | JHDM1D antisense RNA 1 | non-proteing coding RNA |
| -0.52 | -1.13 | | -0.50 | LOC100216546 (LINC01004) | long-intergenic non-protein coding RNA 1004 | non-protein coding RNA |
| -0.37 | -0.30 | | -1.23 | LOC100505666 (DCST1-AS1) | DCST1 antisense RNA 1 | non-protein coding RNA |
| 0.32 | -0.27 | | -1.58 | LOC143666 | uncharacterized LOC143666 | non-protein coding RNA |
| 0.71 | 0.48 | | -1.59 | LOC154761 | family with sequence similarity 115, member C pseudogene | pseudogene |
| -0.76 | -1.39 | | -0.41 | LOC554206 | leucine carboxyl methyltransferase 1 pseudogene | pseudogene |
| 1.02 | 1.02 | | -0.40 | MAFF | MAF bZIP transcription factor F | nucleic acid binding/transcription regulation/stress response |
| 0.48 | 0.08 | | -1.08 | MAFK | MAF bZIP transcription factor K | nucleic acid binding/transcription regulation |
| -0.02 | -0.58 | | -1.01 | MAMDC4 | MAM domain containing 4 | protein transport |
| -1.14 | -0.88 | | -0.56 | MAP2K6 | mitogen-activated protein kinase 6 | cytoskeletal protein/transferase (kinase)/stress response/transcription regulation |
| 0.16 | -0.59 | | -1.00 | MAPK15 | mitogen-activated protein kinase 15 | transferase (kinase) |
| 0.50 | -0.61 | | -1.03 | MEG3 | maternally expressed 3 | non-protein coding RNA/tumor supressor |
| 0.08 | 0.20 | | -1.04 | METRNL | meteorin like, glial cell differentiation regulator | signaling molecule (hormone) |
| 0.50 | -0.22 | | -2.54 | MIR210HG | MIR210 host gene | non-protein coding RNA |
| 0.40 | 0.18 | | -1.53 | MIR4683 | microRNA 4683 | non-protein coding RNA/transcription regulation |
| 1.06 | 0.86 | | -1.53 | MXI1 | MAX interactor 1, dimerization protein | nucleic acid binding/transcription regulation/proto-oncogene |
| 0.27 | -0.25 | | -1.01 | MZF1 | myeloid zinc finger 1 | nucleic acid binding/transcription regulation |
| 0.35 | 0.34 | | -1.00 | NAGS | N-acetylglutamate synthase | transferase (acyltransferase) |
| 1.09 | 0.89 | | -2.46 | NDRG1 | N-myc downstream regulated 1 | cytoskeletal protein/cell adhesion/stress response |
| 0.32 | -0.53 | | -1.26 | NEAT1 | nuclear paraspeckle assembly transcript 1 (non-protein coding) | non-protein coding RNA/transcription regulation/tumor progression |
| 0.65 | 0.50 | | -1.00 | NFIL3 | nuclear factor, interleukin 3 regulated | nucleic acid binding/transcription regulation |
| 1.58 | 1.28 | | 0.39 | NFKBIA | NFKB inhibitor alpha | transcription regulation |
| 1.21 | 1.35 | | 0.77 | NFKBIE | NFKB inhibitor epsilon | transcription regulation |
| 0.45 | 0.24 | | -1.24 | NOG | noggin | developmental protein/signaling molecule |
| 0.88 | 0.70 | | -1.12 | PDGFB | platelet derived growth factor subunit B | developmental protein/signaling molecule (growth factor) |
| 0.65 | 0.53 | | -1.19 | PDK1 | pyruvate dehydrogenase kinase 1 | transferase (kinase)/carbohydrate metabolism |
| 0.70 | 0.33 | | -1.37 | PER1 | period circadian clock 1 | transcription regulation |
| 1.36 | 0.84 | | -1.17 | PFKFB4 | 6-phosphofructo-2-kinase/fructose-2,6-biphosphatase 4 | hydrolase/transferase (kinase) |
| 1.76 | 1.62 | | -1.23 | PIK3IP1 | phosphoinositide-3-kinase interacting protein 1 | cell membrane |
| 0.32 | -0.32 | | -1.27 | PLXNB3 | plexin B3 | receptor |
| 0.98 | 0.21 | | -2.26 | PPFIA4 | PTPRF interacting protein alpha 4 | focal adhesions |
| 0.47 | 0.61 | | -1.09 | PPP1R3C | protein phosphatase 1 regulatory subunit 3C | carbohydrate metabolism |
| 0.31 | 0.23 | | -2.27 | PPP1R3G | protein phosphatase 1 regulatory subunit 3G | carbohydrate metabolism |
| 1.17 | 0.36 | | -0.88 | PPP2R5B | protein phosphatase 2 regulatory subunit B'beta | protein phosphatase regulator |
| 0.52 | -0.58 | | -1.02 | PRRT2 | proline rich transmembrane protein 2 | transmembrane protein/synapse |
| 0.10 | -0.65 | | -1.32 | PRSS53 | protease, serine 53 | hydrolase/protease/signaling molecule |
| 1.39 | 1.06 | | -0.08 | PTGS2 | prostaglandin-endoperoxide synthase 2 | oxidoreductase/peroxidase/inflammatory response |
| 0.33 | 0.20 | | -1.43 | RAB3A | RAB3A, member RAS oncogene family | exocytosis/protein transport |
| 0.69 | 0.46 | | -1.10 | RAB40C | RAB40C, member RAS oncogene family | nucleotide-binding (GTPase)/ubiquitination |
| 0.37 | 0.33 | | -1.10 | RARA | retinoic acid receptor alpha | nucleic acid binding/receptor/transcription regulation/proto-oncogene |
| 0.19 | 0.17 | | -1.12 | RASSF7 | Ras association domain family member 7 | apoptosis/cytoskeleton organization |
| 1.01 | 0.66 | | -2.64 | RCOR2 | REST corepressor 2 | transcription regulation |
| -0.07 | -0.34 | | -1.03 | RHBDL1 | rhomboid like 1 | hydrolase/serine protease |
| 0.75 | 0.77 | | -1.27 | RNF122 | ring finger protein 122 | metal-binding/cell viability |
| 0.39 | -0.09 | | -1.10 | RNF165 | ring finger protein 165 | metal-binding/ligase |
| -1.08 | -0.40 | | -0.25 | SCARA5 | scavenger receptor class A member 5 | ion transport/receptor |
| -1.22 | -0.73 | | 0.23 | SDPR (CAVIN2) | serum deprivation response | lipid-binding |
| 0.71 | 0.30 | | -1.07 | SH3BP2 | SH3 domain binding protein 2 | SH3-binding/signal transduction |
| -0.49 | -1.22 | | -0.32 | SH3BP5-AS1 | SH3BP5 antisense 1 | non-protein coding RNA |
| 1.05 | 0.28 | | -1.07 | SH3D21 | SH3 domain containing 21 | SH3 domain |
| 0.55 | 0.46 | | -1.25 | SLC2A1 | solute carrier family 2 member 1 | sugar transport |
| 1.36 | 1.07 | | -2.28 | SLC2A3 | solute carrier family 2 member 3 | sugar transport |
| 0.15 | 0.02 | | -1.36 | SLC7A5P1 | solute carrier family 7 member 5 pseudogene 1 | amino acid transport |
| 0.26 | -1.06 | | -0.62 | SNORA81 | small nucleolar RNA, H/ACA box 81 | non-protein coding RNA |
| 0.11 | -1.13 | | -0.79 | SNORD52 | small nucleolar RNA, C/D box 52 | non-protein coding RNA |
| 0.34 | -1.02 | | -0.46 | SNORD58C | small nucleolar RNA, C/D box 58C | non-protein coding RNA |
| 0.20 | -0.24 | | -1.74 | SPRY1 | sprouty RTK signaling antagonist 1 | developmental protein |
| 0.30 | -0.01 | | -1.60 | TBX1 | T-box 1 | developmental protein/nucleic acid binding/transcription regulation |
| 1.22 | 0.71 | | 0.03 | TNFAIP3 | TNF alpha induced protein 3 | hydrolase/ligase/inflammatory response/apoptosis |
| 1.07 | 0.84 | | 1.00 | TNIP3 | TNFAIP3 interacting protein 3 | inflammatory response |
| -1.01 | -0.05 | | -0.11 | TSPAN1 | tetraspanin 1 | transmembrane protein/signal transduction |
| 1.63 | 1.78 | | 0.30 | TXNIP | thioredoxin interacting protein | cell cycle/transcription regulation/tumor suppressor |
| 0.31 | -0.40 | | -1.08 | VAMP1 | vesicle associated membrane protein 1 | exocytosis/synapse |
| 0.11 | 0.14 | | -1.02 | VASN | vasorin | signaling molecule |
| 0.72 | 0.53 | | -1.26 | VEGFA | vascular endothelial growth factor A | developmental protein/mitogen/signaling molecule (growth factor) |
| 0.76 | 0.56 | | -1.26 | VLDLR | very low density lipoprotein receptor | lipid metabolism/endocytosis/receptor |
| 0.10 | -0.61 | | -1.06 | YJEFN3 | YjeF N-terminal domain containing 3 | steroid metabolism |
| 0.07 | 0.08 | | -1.39 | ZNF296 | zinc finger protein 296 | metal-binding/nucleic acid binding/transcription regulation |
| 0.55 | 0.39 | | -1.11 | ZNF503 | zinc finger protein 503 | metal-binding/nucleic acid binding/transcription regulation |
| * FDR > 0.01 |  | |  |  |  |  |
|  | upregulated | |  |  |  |  |
|  | downregulated | |  |  |  |  |
|  | not differentially expressed | | | |  |  |
